# Age-dependent diagnostic and correlational architecture of multiplex plasma biomarkers in Alzheimer’s disease: a cross-ethnic, cross-platform validation study

**DOI:** 10.1186/s13195-026-02119-z

**Published:** 2026-06-25

**Authors:** Boru Jin, Aitong Li, Fengling Xu, Cong Chen, Jiawen Dong, Jingjing Wang, Dantong Tang, Ying Gu, Huayan Liu

**Affiliations:** 1 Department of Neurology, First Hospital of China Medical University, Shenyang, Liaoning Province, China; 2 Key Laboratory of Neurological Disease Big Data of Liaoning Province, Shenyang, China; 3 Shenyang Clinical Medical Research Center for Difficult and Serious Diseases of the Nervous System, Shenyang, China

**Keywords:** Plasma p-tau217, Alzheimer’s disease, Early-onset, Late-onset, Age-stratified, Diagnostic accuracy, Biomarker–clinical correlation, Multiplex immunoassay, Cross-ethnic validation, ADNI

## Abstract

**Background:**

Plasma phosphorylated tau-217 (p-tau217) is one of the most accurate blood-based biomarkers for Alzheimer's disease (AD), yet whether its diagnostic and correlational properties vary with age at onset has not been systematically examined.

**Methods:**

We measured eight plasma biomarkers (tau, amyloid, neurodegeneration, neuroinflammation) via automated chemiluminescence immunoassay in a Chinese memory clinic cohort (*n* = 604; amyloid positron emission tomography (PET) reference), with validation in the Alzheimer's Disease Neuroimaging Initiative (ADNI) (*n* = 1,615; amyloid and tau PET). Diagnostic accuracy and biomarker–clinical correlations were compared between early-onset (EO < 65 years) and late-onset (LO ≥ 65 years) subgroups.

**Results:**

Overall accuracy was high (primary area under the curve (AUC) 0.923; ADNI 0.904). In ADNI, p-tau217 showed higher accuracy in EO than LO (AUC 0.940 vs 0.892, *P* = 0.012), most pronounced at age 60 and absent by 70. The optimal biomarker shifted from tau-centric markers in LO to Aβ42/Aβ40 in EO. Tau biomarker–clinical correlations diverged sharply by onset age and were stronger in EO for cognitive and neuropsychiatric outcomes, whereas glial fibrillary acidic protein (GFAP) and neurofilament light chain (NfL) showed no age-dependent heterogeneity. Opposing-direction associations were observed for p-tau217%, which correlated negatively with onset age in AD but positively in Non-AD cognitive impairment. In an exploratory analysis, baseline p-tau217 was associated with longitudinal cognitive decline, numerically greater in EO though based on a small subgroup. Cross-ethnic generalizability was supported by the ADNI-Asian subgroup.

**Conclusions:**

This cross-ethnic, cross-platform study demonstrates that the diagnostic accuracy, biomarker–clinical correlation architecture, and optimal analyte selection of plasma p-tau217 vary systematically with age at onset, most markedly for tau-related measures. These findings challenge universal threshold paradigms and support age-stratified biomarker interpretation in clinical practice and therapeutic trial design.

**Supplementary Information:**

The online version contains supplementary material available at 10.1186/s13195-026-02119-z.

## Background

Plasma phosphorylated tau-217 (p-tau217) has emerged as one of the most accurate blood-based biomarkers for detecting Alzheimer’s disease (AD) amyloid pathology, achieving diagnostic performance comparable to that of cerebrospinal fluid and positron emission tomography (PET) measures [1–3]. Multiplex immunoassay platforms now simultaneously quantify tau, amyloid, neurodegeneration (neurofilament light chain [NfL]), and neuroinflammation (glial fibrillary acidic protein [GFAP]) pathways, offering a multi-pathway window into disease biology [4–6]. These advances carry immediate clinical implications: anti-amyloid immunotherapy eligibility now requires biomarker confirmation of amyloid pathology [7], and the 2024 revised NIA-AA criteria formally integrate blood biomarkers into the diagnostic workup [8]. Beyond binary diagnostic classification, the degree to which biomarker levels track disease severity (cognitive decline, functional impairment, and neuropsychiatric burden) determines their utility for prognosis and individualized counseling [9]. Current interpretation guidelines, however, largely apply unified diagnostic cutoffs without stratification by age at onset [10, 11], a practice that has not been systematically examined.

Converging evidence indicates that early-onset AD (EOAD, symptom onset < 65 years) and late-onset AD (LOAD, ≥ 65 years) differ in clinical presentation and underlying biology. Clinically, EOAD is characterized by distinctive neuropsychiatric profiles (including prominent frontal-behavioral features) and greater phenotypic heterogeneity than LOAD [12–14]. Autopsy, imaging, and biomarker studies further indicate that tau-related pathology and its regional distribution vary across the AD spectrum and with age [15–17], and that older onset is often accompanied by coexisting processes that can complicate how protein markers relate to underlying disease [18, 19]. Age also reshapes the etiological landscape of Non-AD cognitive impairment [20], raising the question of whether biomarker–clinical associations in symptomatic patients are similarly age-dependent.

How these biological differences affect the clinical interpretation of multiplex plasma biomarkers has received limited systematic study. Prior studies suggest that age and clinical context can influence p-tau217 diagnostic performance [3, 21] and that the marker retains utility in young-onset cohorts [22]. Although initial East Asian validation has shown comparable overall performance [23], whether age-stratified biomarker patterns generalize cross-ethnic populations remains untested. Biomarker–neuropsychiatric associations also remain largely unexplored, despite the prominence of behavioral symptoms in EOAD [12, 24]. Critically, no study has directly compared early-onset (EO) and late-onset (LO) diagnostic accuracy, analyte selection, biomarker–severity relationships, or longitudinal predictive value within a unified multicohort framework.

To address these questions, we conducted a cross-ethnic, cross-platform validation study to systematically characterize how the clinical utility of multiplex plasma biomarkers varies with age at onset. We measured eight plasma biomarkers spanning tau, amyloid, neurodegeneration, and neuroinflammation pathways in a primary cohort (*n* = 604) with amyloid PET as the diagnostic reference. We replicated key findings in the Alzheimer's Disease Neuroimaging Initiative (ADNI) cohort (*n* = 1,615), which additionally offered tau PET–based biological anchoring and longitudinal follow-up. Specifically, we compared EO and LO subgroups on diagnostic accuracy, biomarker–clinical severity correlations across cognitive, functional, and neuropsychiatric domains, and the pathway specificity of age-related effects, with Non-AD cognitive impairment as an etiological comparator.

## Methods

### Study design and participants

#### Study setting and overall design

This cross-sectional observational study was conducted in accordance with Standards for Reporting Diagnostic Accuracy Studies (STARD) 2015 guidelines [25] at the memory clinic of the First Hospital of China Medical University (August 2023–July 2025). A total of 604 consecutively recruited participants were included; sample size was determined by the number of eligible participants with available biomarker data during the pre-specified enrollment period. Participants were aged ≥ 50 years, had a reliable caregiver, and had at least one core AD biomarker (^1^⁸F-AV-45 PET or plasma p-tau217) available for etiological classification.

#### Grouping and etiological classification

The normal cognition (NC) group (*n* = 99) consisted of individuals with a global Clinical Dementia Rating (CDR) score of 0. A total of 505 participants with cognitive impairment (CI; CDR ≥ 0.5) were subdivided using the NIA-AA AT(N) framework [26] (Sect. 2.4) into AD (A + T + N +, *n* = 99), Presumed AD (N + with at least one core biomarker positive while the other was unavailable, with consistent clinical features; *n* = 227), and Non-AD CI (*n* = 179). The combined AD group (“AD continuum”) was stratified by age at symptom onset into early-onset (EO < 65 years) and late-onset (LO ≥ 65 years) subgroups; the same cutoff was applied to Non-AD CI. Participants with psychiatric disorders, other neurodegenerative diseases (e.g., Parkinson’s disease, dementia with Lewy bodies [DLB]), recent severe stroke, severe organ dysfunction, or inability to complete assessments were excluded; patients with severe dementia (CDR > 2) were not enrolled (Supplementary Methods). The study was approved by the institutional ethics committee (approval no. [2023] 2023–469-2), with written informed consent obtained from all participants or their legal representatives.

#### Clinical evaluation

Demographic data (age at symptom onset, sex, education, apolipoprotein E (APOE) genotype) were collected for all participants. Standardized neuropsychological testing was administered by trained professionals who were blinded to biomarker and PET results. Assessments included the Mini-Mental State Examination (MMSE) [27], Montreal Cognitive Assessment (MoCA) [28], Clinical Dementia Rating (CDR) and CDR Sum of Boxes (CDR-SB) [29], Activities of Daily Living scale (ADL [30]; with Basic [BADL] and Instrumental [IADL] subscales), Neuropsychiatric Inventory (NPI [31]; frontal-behavioral symptoms were defined as a composite of the agitation/aggression, apathy, disinhibition, irritability, and aberrant motor behavior domain Frequency × Intensity scores), and the Chinese short-form Cohen-Mansfield Agitation Inventory [32] (CMAI-cs), an 18-item caregiver-rated agitation scale with adequate psychometric properties in our cohort (Cronbach’s α = 0.723, ICC = 0.975; detailed validation reported separately).

#### Plasma biomarker assays

Fasting venous blood samples were drawn into EDTA-coated tubes and centrifuged at 2,000 × g for 15 min at 4 °C. Plasma was then aliquoted and stored at − 80 °C, undergoing only a single freeze–thaw cycle before analysis (detailed protocol in Supplementary Methods). Plasma levels of Aβ42, Aβ40, p-tau181, p-tau217, p-tau231, non-phosphorylated tau-217 (np-tau217), NfL, and GFAP were measured using the fully automated Elf S240 Chemiluminescence Immunoassay Analyzer (Vazyme Medical Technology, China) with matched sandwich immunoassay kits (catalog numbers in Supplementary Methods). The derived metric p-tau217% was calculated as p-tau217/(p-tau217 + np-tau217). All assays were performed by personnel blinded to clinical diagnoses, with intra- and inter-assay coefficients of variation < 10% for all analytes. Measurements below the lower limit of quantification were excluded. p-tau231 had a higher rate of missing data as it was assayed only from residual aliquots after all primary measurements were completed.

#### Aβ-PET imaging and diagnostic criteria

Amyloid-PET imaging was performed using ^1^⁸F-florbetapir (AV-45). Scans were independently evaluated by two trained nuclear medicine physicians (inter-rater Cohen’s κ = 0.92, concordance 96.6%; discrepancies resolved by a third senior reader) and classified as amyloid-positive if at least two cortical regions exhibited tracer uptake or one region demonstrated pronounced uptake. Neurodegeneration (N +) was assessed by structural MRI, independently evaluated by a neurologist and neuroradiologist; all CI participants were confirmed N +. Etiological classification followed the AT(N) framework: amyloid (A) status was determined by PET; tau (T +) was defined as plasma p-tau217 > 3.27 pg/mL (manufacturer-recommended threshold). CI participants were classified as AD (A + T + N +), Presumed AD (N + with at least one A + or T + while the other was unavailable, with consistent clinical features), or Non-AD CI (N + without AD biomarker support).

### Statistical analysis

#### Descriptive statistics

Continuous variables, which were non-normally distributed (Shapiro–Wilk test), are summarized as median with interquartile range (IQR); categorical variables are reported as frequencies. Group differences were assessed using the Kruskal–Wallis H test for continuous variables and the chi-square test for categorical variables.

#### Diagnostic performance

Receiver operating characteristic (ROC) analysis was used to assess diagnostic accuracy in participants with ^1^⁸F-AV-45 PET data. Of 206 PET participants, 32 lacked biomarker data, yielding a maximum analytic sample of 174; biomarker-specific sizes varied owing to individual missing values. For each area under the curve (AUC), 95% confidence intervals (CIs) were derived from 1,000 bootstrap resamples, and the optimal cutoff was identified as the point maximizing the Youden Index.

#### Correlation analyses

Spearman’s ρ with Benjamini–Hochberg false discovery rate (FDR) correction was used within the AD continuum (confirmed + Presumed AD, *n* = 326) and Non-AD CI (*n* = 179), justified by moderate subgroup concordance (Pearson r = 0.551, *P* < 0.001). To address potential circularity, patterns were verified in the total CI cohort (*n* = 505) without AT(N)-based subsetting (Supplementary Figure S1). APOE genotypes were converted to a 5-tier ordinal risk score [33]; sex was coded as binary. Correlation pairs with fewer than 10 valid observations were excluded. Analyses were predefined as primary (overall amyloid-PET ROC; overall biomarker–clinical correlations within the AD continuum and Non-AD CI) or exploratory (EO/LO subgroup correlation comparisons and longitudinal analyses) and are labelled accordingly. To further confirm that correlations were not driven by the biomarker-assisted classification of Presumed AD, a sensitivity analysis was repeated in PET-confirmed AD only (*n* = 99) versus Non-AD CI (Supplementary Table S8). To formally test whether age-dependence differed by biomarker class, we fitted for each biomarker–clinical pair an ordinary-least-squares interaction model (standardized clinical ~ standardized biomarker × age group) and compared the standardized interaction coefficients between tau-related and non-tau markers (Supplementary Table S9). Missing data were examined formally (Supplementary Table S6); p-tau231 (55.5% missing) followed a missing-at-random pattern and was excluded from primary inferences.

#### Age-stratified analyses

Spearman correlations were recomputed within EO (symptom onset < 65) and LO ≥ 65 subgroups of the AD continuum (EO *n* = 97, LO *n* = 229) and Non-AD CI (EO *n* = 78, LO *n* = 101). Fisher’s Z-transformation tested EO–LO correlation differences (P_diff).

#### External validation (ADNI)

Independent validation used the Alzheimer’s Disease Neuroimaging Initiative [34] (ADNI; *n* = 1,615; 847 cognitively normal [CN], 579 mild cognitive impairment [MCI], 186 dementia [DEM], and 3 with missing baseline diagnosis). Plasma p-tau217 (Lilly immunoassay) and Aβ42/Aβ40 (Lumipulse) were assessed against amyloid PET positivity (Centiloid ≥ 20.6, per ADNI processing pipeline); tau PET standardized uptake value ratios (SUVRs) used ^1^⁸F-flortaucipir. As ADNI does not record symptom-onset age, baseline age was used to stratify EO (< 65, *n* = 335) and LO (≥ 65, *n* = 1,280) groups. DeLong’s test compared EO vs LO AUCs; sensitivity analyses used multiple cutpoints (55, 60, 65, 70 years) and a grey-zone analysis excluding ages 60–70. Post-hoc power was estimated for the primary cohort comparison. Longitudinal analysis assessed baseline p-tau217 versus annualized cognitive decline rates. Cross-ethnic generalizability was evaluated in the ADNI Asian subgroup.

Analyses were performed in SPSS 26.0 and Python 3.9. Statistical significance was set at two-sided *P* < 0.05.

## Results

### Demographic, clinical, and biomarker characteristics

A total of 604 participants were included: 99 NC and 505 CI, subdivided into AD (*n* = 99), Presumed AD (*n* = 227), and Non-AD CI (*n* = 179) (Table 1). The NC reference group (median age 61.0 [IQR 56.0–67.5] years; education 12.0 [9.0–14.0] years) anchored the chemiluminescence assay baseline, while the CI cohort mirrored typical memory clinic demographics (median symptom onset 68.0 [62.0–74.0] years; education 9.0 [7.0–12.0] years; both *P* < 0.001 vs NC). Sex distribution did not differ across groups (*P* = 0.603). APOE ε4 carrier rates were higher in AD (57.7%) and Presumed AD (58.7%) than in NC (14.8%) and Non-AD CI (21.8%; *P* < 0.001). Clinical severity worsened progressively from NC through Non-AD CI to the AD continuum (Table 1). Plasma biomarker profiles showed expected pathobiological patterns: tau biomarkers and GFAP were elevated in the AD continuum, while Aβ42/Aβ40 was reduced (all *P* < 0.001); Presumed AD closely paralleled confirmed AD across all analytes, supporting the presumed Alzheimer’s etiology. Agitation profiles (CMAI-cs) showed no significant EO–LO differences in either the AD continuum or Non-AD CI (all *P* > 0.05), suggesting that the age-dependent biomarker divergences described below are not secondary to differing symptomatic severities.

**Table 1:** Demographic, clinical, and plasma biomarker characteristics of the study cohort

Characteristics	NC (*n* = 99)	Total CI (*n* = 505)	AD (*n* = 99)	Presumed AD (*n* = 227)	Non-AD CI (*n* = 179)	*P* value
N	99	505	99	227	179	
Demographics						
Age, years	61.0 (56.0–67.5)	68.0 (62.0–74.0)	68.0 (61.0–73.0)	70.0 (64.0–75.0)	66.0 (61.0–70.0)	< 0.001
Education, years	12.0 (9.0–14.0)	9.0 (7.0–12.0)	9.0 (9.0–12.0)	9.0 (7.0–11.0)	9.0 (7.0–12.0)	< 0.001
Female, n/N (%)	73/99 (73.7)	358/505 (70.9)	67/99 (67.7)	167/227 (73.6)	124/179 (69.3)	0.603
APOE ε4 carrier, n/N (%)	12/81 (14.8)	218/480 (45.4)	56/97 (57.7)	125/213 (58.7)	37/170 (21.8)	< 0.001
Clinical Assessments						
CDR, n (%)						< 0.001
0.5	—	266 (52.7)	36 (36.4)	93 (41.0)	137 (76.5)	
1	—	150 (29.7)	42 (42.4)	78 (34.4)	30 (16.8)	
2	—	89 (17.6)	21 (21.2)	56 (24.7)	12 (6.7)	
CDR-SB	0.0 (0.0–0.0)	4.0 (2.5–6.5)	5.0 (3.5–7.5)	5.0 (3.0–8.0)	3.0 (2.0–4.5)	< 0.001
MMSE	28.0 (27.0–30.0)	22.0 (18.0–25.0)	21.0 (17.0–24.0)	21.0 (16.0–24.0)	25.0 (22.0–27.0)	< 0.001
MoCA	25.0 (23.0–27.0)	17.0 (13.0–20.0)	16.0 (12.0–19.0)	15.0 (11.0–18.0)	19.0 (16.0–22.0)	< 0.001
BADL	8.0 (8.0–8.0)	8.0 (8.0–8.0)	8.0 (8.0–8.0)	8.0 (8.0–8.0)	8.0 (8.0–8.0)	< 0.001
IADL	12.0 (12.0–12.0)	15.0 (13.0–20.0)	16.0 (14.0–21.0)	16.0 (13.0–22.2)	13.0 (12.0–15.0)	< 0.001
ADL (BADL + IADL)	20.0 (20.0–20.0)	23.0 (21.0–28.0)	24.0 (22.0–29.2)	24.0 (21.0–31.0)	21.0 (20.0–23.0)	< 0.001
Plasma Biomarkers						
p-tau181, pg/mL	1.8 (1.3–2.2)	3.8 (2.1–5.7)	5.0 (4.0–6.8)	5.1 (3.8–6.6)	1.8 (1.3–2.3)	< 0.001
p-tau217, pg/mL	1.9 (1.4–2.4)	4.7 (2.1–7.1)	7.0 (5.3–8.9)	6.2 (4.9–8.6)	1.7 (1.2–2.3)	< 0.001
Aβ42, pg/mL	7.1 (6.1–8.3)	6.5 (5.6–7.5)	5.9 (5.3–6.7)	6.1 (5.3–7.0)	7.3 (6.3–8.3)	< 0.001
Aβ42/Aβ40	0.0652 (0.0610–0.0698)	0.0540 (0.0496–0.0622)	0.0520 (0.0481–0.0549)	0.0516 (0.0478–0.0552)	0.0631 (0.0566–0.0676)	< 0.001
GFAP, pg/mL	73.0 (47.1–91.8)	138.0 (89.0–194.2)	163.2 (132.1–216.0)	173.5 (133.4–229.2)	85.3 (61.3–110.3)	< 0.001
NfL, pg/mL	27.7 (21.6–45.7)	46.5 (33.9–65.0)	47.1 (37.0–60.0)	52.2 (36.5–71.5)	37.2 (25.7–56.6)	< 0.001

Demographic, clinical, and plasma biomarker characteristics of 604 consecutively recruited participants stratified by etiological classification: normal cognition (NC, *n* = 99), confirmed AD (A + T +, *n* = 99), Presumed AD (*n* = 227), and Non-AD cognitive impairment (*n* = 179). Age at symptom onset, education, and all clinical/biomarker variables are presented as median (IQR). Sex, APOE ε4 carrier status, and CDR grade are presented as n (%). *P* values were calculated using the Kruskal–Wallis test for continuous variables and the chi-square test for categorical variables across all four groups

*AD* Alzheimer's disease, *CDR* Clinical Dementia Rating, *CDR-SB* CDR Sum of Boxes, *CI* cognitive impairment, *GFAP* glial fibrillary acidic protein, *MMSE* Mini-Mental State Examination, *MoCA* Montreal Cognitive Assessment, *NfL* neurofilament light chain; *p-tau* phosphorylated tau

### Diagnostic performance and age-dependent divergence

**Fig. 1:**
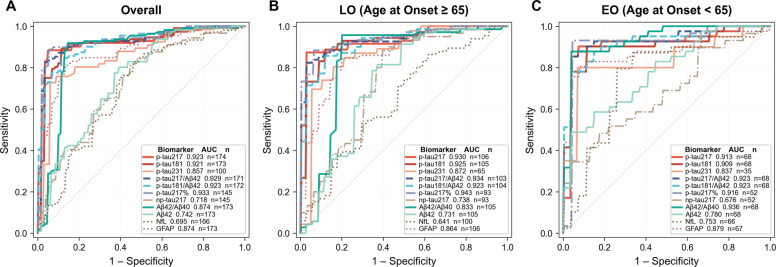
Diagnostic performance of chemiluminescence plasma biomarkers for predicting amyloid positivity. Receiver operating characteristic (ROC) curves illustrate the discriminative accuracy of plasma biomarkers against Aβ-PET status. **A** Overall validation sub-cohort (analytic *n* = 100–174 depending on biomarker availability). **B** Late-onset (LO) sub-cohort (analytic *n* = 65–106). **C** Early-onset (EO) sub-cohort (analytic *n* = 35–68). Abbreviations: AUC, area under the curve; CI, confidence interval; EO, early-onset (symptom onset < 65 years); LO, late-onset (symptom onset ≥ 65 years)

Of 206 participants with Aβ-PET imaging, 174 had concurrent biomarker data (66–68 EO, 100–106 LO; Fig. 1, Table 2). The chemiluminescence platform achieved high discriminative accuracy for amyloid positivity: p-tau217% yielded the highest AUC (0.933), followed by p-tau217/Aβ42 (0.929) and p-tau217 (0.923). Empirically derived optimal cutoffs closely matched commercial thresholds (e.g., Youden-optimal p-tau217: 3.29 vs commercial 3.27 pg/mL). Across top-performing tau-centric markers, specificity consistently exceeded sensitivity (e.g., p-tau217%: sensitivity 0.89, specificity 0.95; Supplementary Table S1), reflecting the clinical composition (CDR 0.5–2.0) that mirrors real-world memory clinic populations, thereby enhancing the ecological validity of the derived thresholds.

**Table 2:** Diagnostic performance metrics and optimal cutoffs of plasma biomarkers against Aβ-PET status

Biomarker	Overall N	Overall AUC (95% CI)	Overall Se	Overall Sp	Overall Cutoff	EO N	EO AUC (95% CI)	EO Se	EO Sp	LO N	LO AUC (95% CI)	LO Se	LO Sp
p-tau217	174	0.923 (0.875–0.967)	0.88	0.95	3.291	68	0.913 (0.823–0.983)	0.9	0.93	106	0.93 (0.874–0.972)	0.87	0.97
p-tau181	173	0.921 (0.877–0.960)	0.89	0.89	2.65	68	0.909 (0.823–0.976)	0.9	0.89	105	0.925 (0.868–0.973)	0.89	0.88
p-tau231	100	0.857 (0.768–0.929)	0.76	0.91	2.91	35	0.837 (0.684–0.966)	0.8	0.93	65	0.872 (0.749–0.961)	0.8	0.84
p-tau217/Aβ42	171	0.929 (0.888–0.966)	0.83	0.97	0.615	68	0.923 (0.841–0.986)	0.85	0.96	103	0.934 (0.885–0.974)	0.82	0.97
p-tau181/Aβ42	172	0.923 (0.879–0.958)	0.85	0.89	0.454	68	0.923 (0.849–0.979)	0.78	0.96	104	0.923 (0.872–0.969)	0.86	0.88
p-tau217%	145	0.933 (0.888–0.973)	0.89	0.95	0.011	52	0.916 (0.808–1.000)	0.93	0.96	93	0.943 (0.895–0.980)	0.88	0.94
np-tau217	145	0.718 (0.633–0.801)	0.81	0.55	295.105	52	0.676 (0.529–0.811)	0.48	0.83	93	0.738 (0.620–0.845)	0.88	0.61
Aβ42/Aβ40	173	0.874 (0.805–0.937)	0.92	0.85	0.059	68	0.936 (0.866–0.986)	0.88	0.96	105	0.833 (0.726–0.929)	0.96	0.8
Aβ42	173	0.742 (0.662–0.822)	0.8	0.61	6.894	68	0.78 (0.673–0.879)	0.49	0.96	105	0.731 (0.620–0.838)	0.79	0.66
NfL	166	0.695 (0.611–0.779)	0.83	0.54	35.224	66	0.753 (0.627–0.878)	0.87	0.67	100	0.641 (0.522–0.757)	0.86	0.38
GFAP	173	0.874 (0.820–0.926)	0.81	0.87	111.528	67	0.879 (0.786–0.952)	0.8	0.92	106	0.864 (0.781–0.936)	0.85	0.8

Receiver operating characteristic analysis of chemiluminescence-based plasma biomarkers for discriminating amyloid PET-positive from PET-negative participants. Results are presented for the overall validation sub-cohort and stratified by age at symptom onset (early-onset [EO] < 65 years; late-onset [LO] ≥ 65 years). *N* = analytic sample size after exclusion of participants missing the specific biomarker; *AUC* area under the receiver operating characteristic curve (95% CI by 1,000 bootstrap resamples); *Se* sensitivity; *Sp* specificity at the empirically derived optimal cutoff maximizing the Youden Index. Cutoff units: pg/mL for individual analytes, ratio for composite indices, % for p-tau217%

Stratifying by age revealed divergent performance: in the LO subgroup, tau-centric markers dominated, with p-tau217% achieving the highest AUC (0.943) and p-tau217 alone reaching 0.930, while in the EO subgroup, the Aβ42/Aβ40 ratio emerged as the strongest predictor (AUC = 0.936), outperforming individual p-tau measurements (p-tau217 AUC = 0.913). These results indicate that the optimal blood-based proxy for AD pathology differs by age at symptom onset.

### Biomarker–clinical correlation mapping

**Fig. 2:**
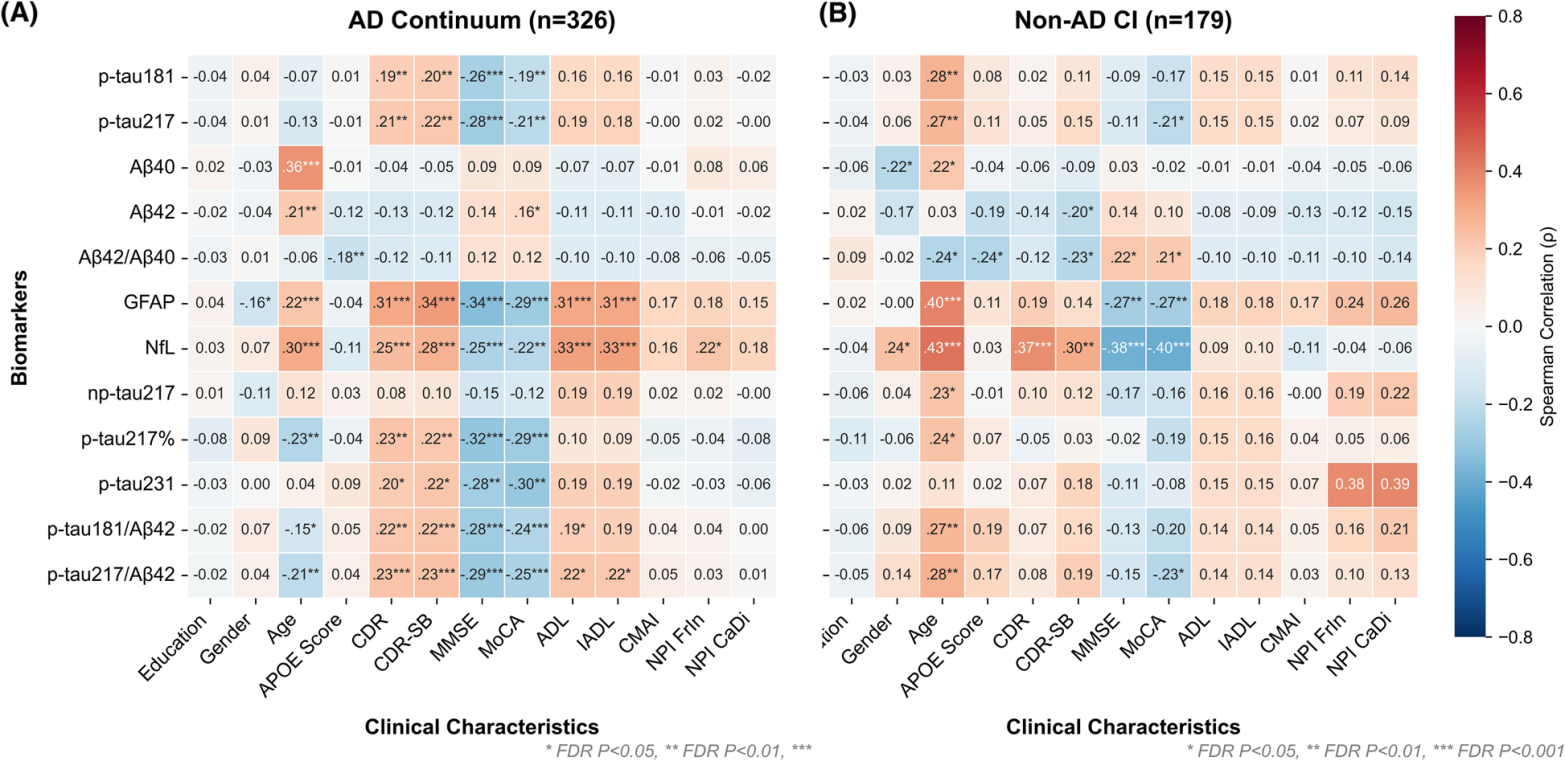
Biomarker–clinical correlation heatmaps in the Alzheimer's disease continuum and non-Alzheimer's cognitive impairment. Heatmaps display Spearman rank correlation coefficients between plasma biomarkers (rows) and clinical characteristics (columns) within the AD continuum. (**A**) AD continuum (n = 326); (**B**) Non-AD CI group (n = 179). P-values adjusted using the Benjamini–Hochberg FDR method. Significance levels: * FDR *P* < 0.05, ** FDR *P* < 0.01, *** FDR *P* < 0.001. Color scale: Spearman ρ from − 0.8 (blue) to + 0.8 (red). NPI FrI*n* = Neuropsychiatric Inventory Frequency × Intensity; NPI CaDi = Neuropsychiatric Inventory Caregiver Distress

Spearman correlations were performed within the AD continuum (*n* = 326) and Non-AD CI (*n* = 179), with patterns verified in the total CI cohort (Supplementary Figure S1; Fig. 2, Supplementary Table S2; subgroup-specific heatmaps in Supplementary Figure S2). Within the AD continuum, tau biomarkers correlated with cognitive measures (p-tau217 vs MMSE: ρ = − 0.283, *P* < 0.001; vs MoCA: ρ = − 0.213, *P* < 0.001; vs CDR-SB: ρ = 0.215, *P* < 0.001), while GFAP was the strongest severity correlate (CDR-SB: ρ = 0.340, MMSE: ρ = − 0.338; both *P* < 0.001). In Non-AD CI, tau showed minimal cognitive associations, while NfL was the primary decline correlate (MMSE: ρ = − 0.383, MoCA: ρ = − 0.398; both *P* < 0.001). An opposing-direction association emerged: p-tau217% correlated negatively with symptom-onset age in the AD continuum (ρ = − 0.232, P < 0.001) but positively in Non-AD CI (ρ = + 0.235, P = 0.006), suggesting that elevated tau in younger AD patients may reflect intrinsic disease aggressiveness rather than aging.

### Age-stratified biomarker–clinical correlations

**Fig. 3:**
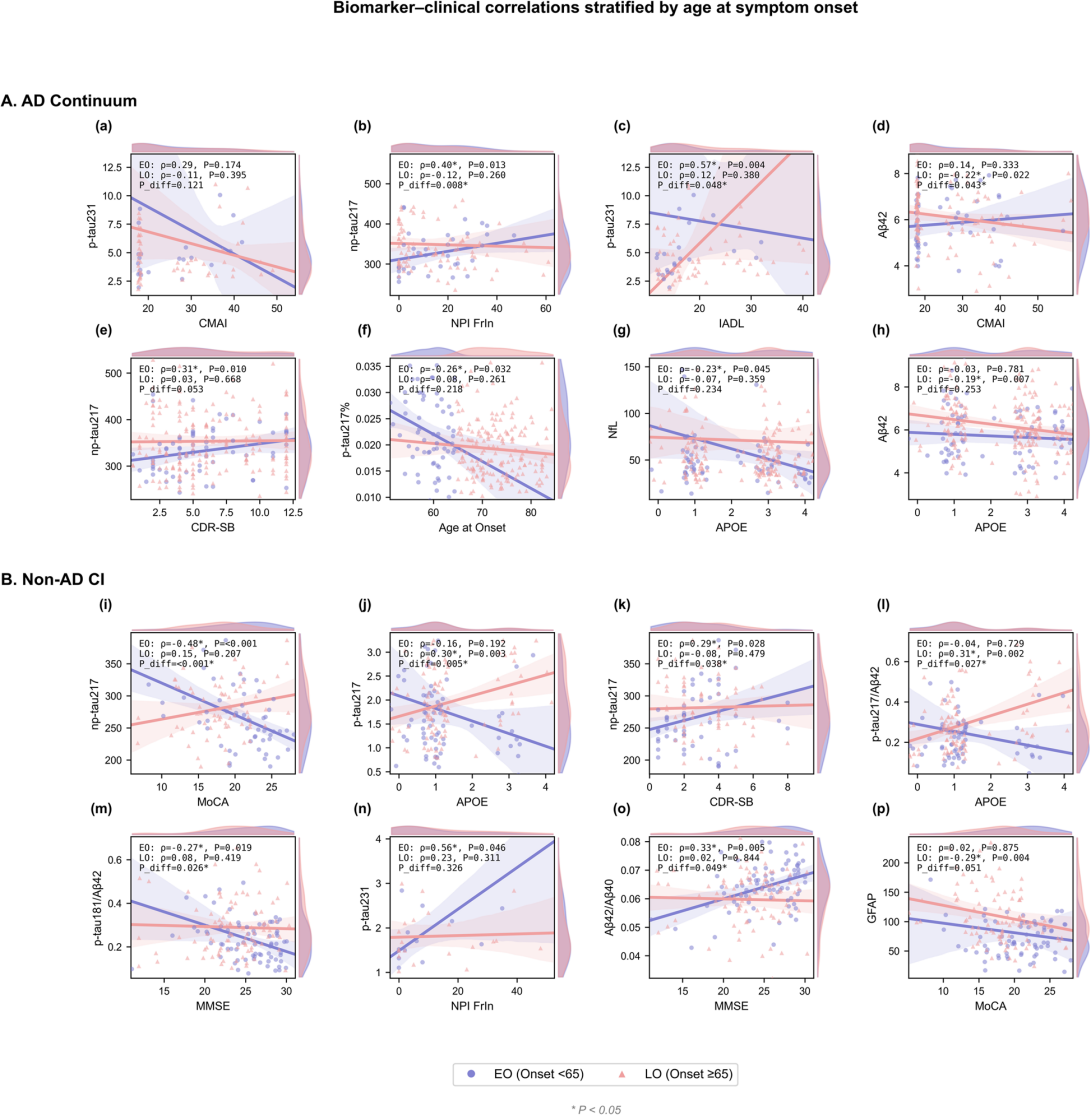
Biomarker–clinical correlations stratified by age at symptom onset. **A** AD continuum (EO *n* = 97, LO *n* = 229). **B** Non-AD CI (EO *n* = 78, LO *n* = 101). EO (purple circles), LO (pink triangles), each with a regression line and shaded 95% confidence band. Each panel reports the Spearman ρ effect size for both subgroups, raw P, and Fisher's Z-test for the difference between EO and LO correlations (*P*_diff); * *P* < 0.05. Effect-size estimates with 95% confidence intervals for the age-group interaction are provided in Supplementary Table S9. Full parameters including FDR-adjusted P values in Supplementary Table S3

Correlations were stratified by symptom-onset age (AD continuum: EO *n* = 97, LO *n* = 229; Non-AD CI: EO *n* = 78, LO *n* = 101; Fig. 3, Supplementary Table S3; complete scatter plots in Supplementary Figure S3). Within the AD continuum, several relationships showed significant age-dependent heterogeneity: the Aβ42–CMAI association diverged between age groups (EO ρ = + 0.14 vs LO ρ = − 0.22, P_diff = 0.043), and EO patients showed markedly stronger np-tau217–NPI correlations for both frontal symptoms (EO ρ = + 0.41 vs LO ρ = − 0.12, P_diff = 0.008) and caregiver distress (P_diff = 0.019). Within Non-AD CI, age differences were more pronounced: np-tau217–MoCA diverged markedly (EO ρ = − 0.48 vs LO ρ = + 0.15, P_diff < 0.001), and the APOE–p-tau217 association was LO-specific (P_diff = 0.005), suggesting that the drivers of tau accumulation and their cognitive consequences may differ between EO and LO patients. In contrast, no significant age-dependent heterogeneity was observed for GFAP or NfL correlations within either diagnostic group (all P_diff > 0.05; Supplementary Table S3), consistent with an age-dependence that was more pronounced for tau-related markers. In a sensitivity analysis restricted to PET-confirmed AD (*n* = 99), the direction of these age-stratified associations was preserved (concordant for 7 of 8 headline pairs and for 93–100% of pairs with a non-negligible effect size; Supplementary Table S8), although statistical significance was attenuated by the smaller sample (median post-hoc power 22%). A formal interaction analysis showed that tau-related markers exhibited age-dependent interactions more often than non-tau markers (19% vs 7%; Fisher *P* = 0.028), an effect driven by tau–amyloid composite ratios; single-analyte tau markers alone did not differ from non-tau markers (Supplementary Table S9).

### External validation in the ADNI cohort

**Fig. 4:**
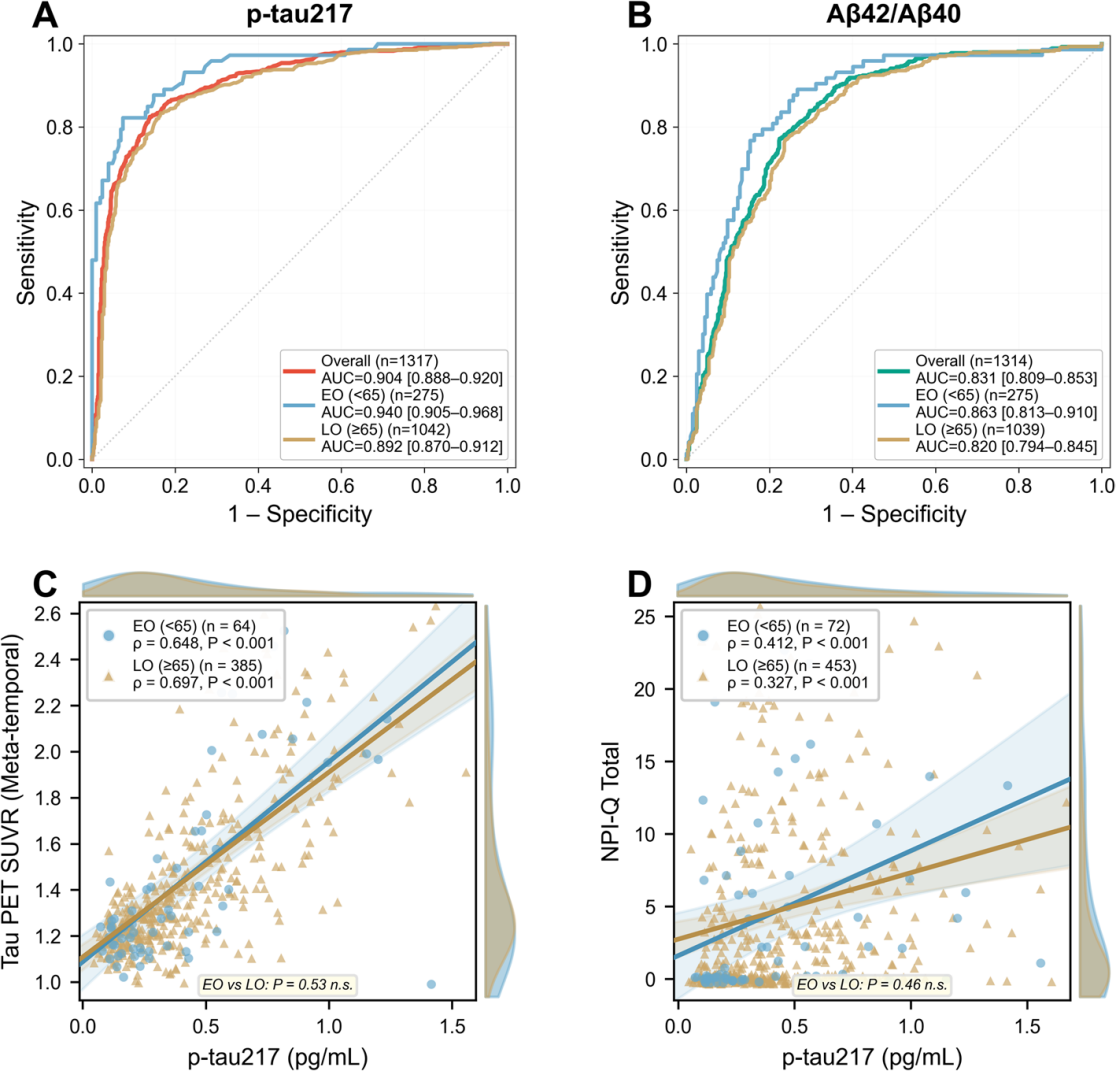
External validation of diagnostic performance and biomarker–clinical correlations in the ADNI cohort. **A** ROC curve for plasma p-tau217 predicting amyloid PET positivity (*n* = 1,317). Overall AUC = 0.904; EO AUC = 0.940; LO AUC = 0.892. DeLong's *P* = 0.012. **B** ROC curve for plasma Aβ42/Aβ40 ratio (*n* = 1,314). Overall AUC = 0.831. DeLong's *P* = 0.11. **C** Scatter plot of plasma p-tau217 vs tau PET SUVR (meta-temporal) within the Aβ + subgroup (*n* = 449). **D** Scatter plot of plasma p-tau217 vs NPI-Q Total Score within the Aβ + subgroup (*n* = 525). EO (blue circles), LO (amber triangles). Fisher's Z-test P values for EO vs LO correlation difference shown at bottom of each panel

Independent validation used the ADNI database (*n* = 1,615; Supplementary Table S7). Among 1,317 participants with concurrent plasma p-tau217 and amyloid PET data, overall diagnostic accuracy was high (AUC = 0.904), consistent with our primary cohort (AUC = 0.923; Fig. 4A). Plasma Aβ42/Aβ40 showed similar cross-cohort performance (ADNI AUC = 0.831 vs primary 0.874; Fig. 4B).

#### Age-stratified performance

DeLong’s test showed that p-tau217 achieved higher accuracy in EO than LO (AUC = 0.940 vs 0.892, *P* = 0.012; Fig. 4A); this advantage was absent for Aβ42/Aβ40 (*P* = 0.11). The primary cohort showed no significant EO–LO difference (DeLong *P* = 0.72), likely reflecting insufficient power in the smaller PET-verified subgroup.

#### Sensitivity analyses

The grey-zone analysis (excluding ages 60–70) confirmed the EO advantage (< 60 AUC = 0.968 vs ≥ 70 AUC = 0.902, *P* < 0.001; Supplementary Table S4). In the ADNI Asian subgroup (*n* = 75), p-tau217 achieved AUC = 0.895, matching the primary Chinese cohort. Multi-cutpoint analysis revealed a clear age gradient: the EO advantage was most pronounced at age 60 (ΔAUC = + 0.070, *P* < 0.001), significant at 65 (*P* = 0.012), and absent at 70 (*P* = 0.59); this gradient was absent for Aβ42/Aβ40 (all *P* > 0.10; Supplementary Figure S4). Youden-optimal thresholds differed between age groups (EO cutoff ~ 11% lower than LO, with higher specificity [0.926 vs 0.834]); note that these ADNI thresholds (Lilly immunoassay) are platform-specific and not directly comparable to the Vazyme chemiluminescence cutoffs reported for our primary cohort. Nonetheless, the marked divergence underscores the clinical risk of applying a single universal threshold.

#### Correlation and PET validation

Within the ADNI Aβ + subgroup (*n* = 541), plasma p-tau217 correlated with MMSE (EO ρ = − 0.610, LO ρ = − 0.483), CDR-SB (EO ρ = 0.718, LO ρ = 0.626), and regional tau PET SUVRs including meta-temporal (EO ρ = 0.648, LO ρ = 0.697; Fig. 4C), entorhinal (EO ρ = 0.600, LO ρ = 0.622), and amygdala regions (EO ρ = 0.688, LO ρ = 0.558; Supplementary Figure S5). NPI-Q scores similarly correlated with p-tau217 (Fig. 4D), demonstrating that age-stratified plasma associations accurately reflect brain tau pathology.

#### Longitudinal prediction

Among 339 ADNI participants (median follow-up 4.0 years), baseline p-tau217 predicted cognitive decline (MMSE: ρ = − 0.54; CDR-SB: ρ = 0.56; both *P* < 0.001; Supplementary Table S5). Within the Aβ + subgroup (*n* = 107), the predictive value appeared greater in EO patients (ΔCDR-SB/year: EO ρ = 0.818, *n* = 14 vs LO ρ = 0.599, *n* = 88; 5 LO participants lacked usable longitudinal CDR-SB slopes), although the small EO sample precludes firm conclusions.

## Discussion

In this multiplex, cross-ethnic, cross-platform validation study (employing a fully automated chemiluminescence immunoassay platform), we report three principal findings. First, the diagnostic accuracy of plasma p-tau217 for amyloid positivity follows a systematic age gradient, as demonstrated in the well-powered ADNI cohort. Second, the correlational architecture linking tau biomarkers to cognitive and neuropsychiatric severity differs between EOAD and LOAD. Third, this divergence was most pronounced for tau-related measures (particularly tau–amyloid composite ratios) against a backdrop of age-stable non-tau reference markers, with opposing-direction associations between AD and Non-AD populations. Together, these findings challenge the assumption that blood-based biomarker interpretation can be age-invariant [8, 11].

Despite robust overall accuracy in both cohorts (primary AUC 0.923; ADNI AUC 0.904), a clear age gradient emerged when diagnostic performance was stratified by onset age. The well-powered ADNI cohort revealed a significant EO advantage (AUC 0.940 vs 0.892, *P* = 0.012). Our primary cohort did not replicate this difference (DeLong *P* = 0.72), likely reflecting its smaller PET-verified sample and limited statistical power (post-hoc power = 23.3%). Although prior work has noted reduced p-tau217 accuracy in the oldest adults [3], age-dependent interpretation in MCI [21], and diagnostic performance in early-onset populations [22], none has directly compared EO versus LO accuracy within a multicohort framework [1, 2], a gap the present study was designed to address. This gradient proved robust across multiple age cutpoints and was absent for the Aβ42/Aβ40 ratio, underscoring its predominantly tau-related nature [35]; cross-ethnic generalizability was further supported by comparable performance between our Chinese primary cohort and the ADNI-Asian subgroup (AUC 0.895) [23]. The primary cohort further revealed that the optimal single biomarker shifted by age, moving from tau-centric markers in LO (p-tau217/Aβ42 AUC 0.934) to the Aβ42/Aβ40 ratio in EO (AUC 0.936), suggesting that the age effect extends beyond accuracy magnitude to diagnostic analyte selection. Consistent with this, Youden-optimal thresholds for p-tau217 were 11% lower in the EO group, with higher specificity (0.926 vs 0.834). Overall, these results indicate that the age effect on p-tau217 diagnostic utility is not merely quantitative but qualitative, extending to analyte selection and decision thresholds, and arguing for age-contextualized interpretation in clinical practice.

Our multiplex panel, paired with a broad neuropsychiatric battery (NPI, CMAI), revealed that tau-specific biomarker–clinical correlations also diverged sharply by onset age. Within the AD continuum, associations between plasma tau and cognitive-neuropsychiatric severity that were strongly evident in EO often reversed direction or vanished in LO. For example, EO patients showed markedly stronger tau–NPI correlations for frontal-behavioral symptoms (EO ρ = + 0.41 vs LO ρ = − 0.12, *P_diff* = 0.008), whereas these associations were absent or reversed in LO, a pattern consistent with the qualitatively distinct neuropsychiatric profile of EOAD [12, 24]. Within Non-AD CI, age-based heterogeneity was even more pronounced: the np-tau217–MoCA association reversed sign (EO ρ = − 0.48 vs LO ρ = + 0.15, *P_diff* < 0.001), likely reflecting distinct etiological compositions, FTD enrichment in younger patients [20] versus vascular/LATE co-pathology in older patients [18]. Consistent with this etiological heterogeneity, a late-onset-specific APOE–tau correlation within this group (*P_diff* = 0.005) suggests that APOE may potentiate tau accumulation through age-dependent pathways in older Non-AD individuals [36, 37]; however, this exploratory observation awaits independent replication. Critically, the ADNI cohort provided independent biological validation: amyloid PET anchored the diagnostic gradient (P1), while regional tau PET SUVRs confirmed the direction of these age-stratified clinical associations [38, 39], demonstrating that peripheral biomarker dynamics capture the underlying brain tau topography. Collectively, these divergent correlation patterns indicate that the biological significance of elevated plasma tau may differ between age groups, a pattern we interpret as consistent with more tau-dominant pathology in EO versus greater multi-pathway contributions in LO, with implications for age-stratified biomarker interpretation.

The predominance of these age effects among tau-related markers is underscored by the behavior of non-tau reference biomarkers. Within our multiplex landscape, GFAP strongly tracked global disease severity (MMSE, CDR-SB) across the AD continuum [40], while NfL was the primary correlate of cognitive decline in the Non-AD CI group, patterns consistent with established neurodegenerative frameworks [41]. When correlations were stratified by age, however, neither GFAP nor NfL showed significant age-dependent heterogeneity in either diagnostic group (all *P_diff* > 0.05; Supplementary Table S3). Against this stable backdrop, the unique age-dependent divergence of tau biomarkers becomes particularly salient. An opposing-direction association further supports this pattern: p-tau217% correlated negatively with age at symptom onset within the AD continuum (ρ = − 0.232, *P* < 0.001) but positively in the Non-AD CI group (ρ = + 0.235, *P* = 0.006). This opposing pattern suggests that elevated plasma tau in younger AD patients may reflect intrinsic disease aggressiveness [16, 17] rather than a general aging effect.

We hypothesize that these divergent biomarker profiles may reflect different predominant disease drivers; we present this as an interpretive model rather than a conclusion established by the present data. EOAD is increasingly conceptualized as a purer, more aggressive tauopathy characterized by rapid trans-synaptic spread and higher intrinsic tau burden [38, 39, 42, 43]. This predominant tau drive yields a stronger, less confounded signal that translates into superior diagnostic discrimination (higher AUC) and creates tighter, more linear mappings between tau biomarkers and clinical severity. Conversely, LOAD is typically driven by a convergence of age-related co-pathologies, including limbic-predominant age-related TDP-43 encephalopathy (LATE), cerebrovascular disease, and neuroinflammation [18, 19]. These multiple interacting pathways dilute the tau-specific diagnostic signal, while leaving non-tau markers unaffected, and attenuate the linear relationship between tau biomarkers and clinical manifestations, as cognitive decline is no longer solely dependent on tau accumulation. The behavioral similarity observed between our age subgroups (via CMAI-cs) demonstrates that these biomarker divergences are not secondary artifacts of differing symptomatic severities, but instead reflect distinct pathological trajectories. Together, this dual-pathway model offers one plausible interpretation for the concurrent divergence of diagnostic accuracy and biomarker–clinical correlations across age groups; direct mechanistic validation, including neuropathological and genetic characterization, will be required to test it.

These age-dependent findings carry direct implications for clinical translation. The multi-cutpoint sensitivity analysis revealed that the EO diagnostic advantage followed a clear gradient, most pronounced at the age-60 cutpoint (ΔAUC = + 0.070, *P* < 0.001) and vanishing entirely by age 70 (*P* = 0.59). This indicates that age-stratified thresholds would primarily benefit the youngest patients, where the clinical need is greatest. Applying a universal, typically older-skewed threshold may systematically disadvantage younger patients (those with aggressive tauopathy who are already prone to delayed diagnosis) by increasing their risk of false negatives [13]. These patterns challenge the age-invariant framework that currently underpins blood biomarker interpretation guidelines [10]. The correlational divergence carries additional clinical significance: in early-onset patients, elevated tau signals more aggressive cognitive and behavioral decline, whereas in late-onset patients the same elevation confers less predictive weight for neuropsychiatric outcomes, a distinction with direct relevance to individualized patient counseling and neuropsychiatric symptom management. Beyond diagnosis, an exploratory longitudinal analysis found that baseline p-tau217 was associated with future functional decline; although this association appeared numerically greater in early-onset patients (CDR-SB ρ = 0.818, *n* = 14 vs LO ρ = 0.599, *n* = 88), the very small early-onset subgroup (*n* = 14) precludes any firm prognostic claim, and this observation requires replication in larger longitudinal cohorts. For biomarker-guided therapeutic trials, these findings suggest that age-stratified enrollment criteria and age-specific biomarker thresholds may reduce misclassification and improve trial efficiency [44]. In addition, the present study demonstrates that a fully automated chemiluminescence immunoassay platform achieves diagnostic accuracy for p-tau217% (AUC 0.933) within the range reported for established platforms [3]. This suggests that p-tau217 testing may be feasibly deployed in routine clinical laboratories without specialized instrumentation, a critical step toward scalable, equitable biomarker access across resource-diverse healthcare settings.

Several limitations warrant consideration. First, the diagnosis of the full ‘Presumed AD’ group relied on a plasma p-tau217 threshold, potentially introducing restricted-range bias; however, the correlation patterns were reproduced in the ungrouped total CI cohort (*n* = 505; Supplementary Figure S1) and supported by moderate concordance with confirmed AD (r = 0.551, *P* < 0.001; Supplementary Figure S2). Second, missing data for p-tau231 (55.5%) followed a missing-at-random pattern (Supplementary Table S6); importantly, the primary diagnostic and correlational analyses were based on np-tau217 and p-tau217, which had near-complete data. Third, our primary cohort encompassed CDR 0.5–2.0 without preclinical or severe stages, which may account for the sensitivity–specificity asymmetry but accurately mirrors the clinical population seeking etiologic workup. Fourth, the primary cohort’s cross-sectional, single-center design limits causal inference; age definitions varied between cohorts (symptom onset vs baseline age), though the grey-zone analysis supported robustness. Fifth, the primary cohort used a fully automated chemiluminescence immunoassay platform (Elf S240, Vazyme) with limited independent validation for plasma p-tau217; however, its diagnostic performance (p-tau217% AUC 0.933) was comparable to established platforms (Lumipulse AUC 0.93–0.96; Simoa AUC 0.92–0.96), and the cross-platform ADNI validation supported generalizability. Finally, genetic characterization was limited to APOE genotyping, precluding identification of specific genetic risk variants beyond APOE; moreover, the APOE–tau interaction was observed only in the Non-AD CI subgroup and requires independent replication. In addition, the EO/LO subgroup correlation comparisons were exploratory and based on modest, imbalanced subgroups; a sensitivity analysis restricted to PET-confirmed AD preserved the direction of these associations but with reduced statistical power (Supplementary Table S8). Finally, because EO/LO status was defined by symptom-onset age in the primary cohort but by baseline age in ADNI (non-equivalent constructs), cross-cohort comparisons should be regarded as complementary rather than as direct replication.

To our knowledge, this is the first study to systematically demonstrate both an age-dependent diagnostic gradient and age-divergent biomarker–clinical correlation patterns for plasma p-tau217, a divergence most pronounced for tau-related measures, interpreted through a hypothesized pathobiological framework. The multi-dimensional validation, spanning ethnicities, assay platforms, and assessment modalities (clinical scores and tau PET), reinforces generalizability.

## Conclusions

Plasma p-tau217 demonstrates higher diagnostic accuracy and different biomarker–clinical associations in early-onset compared with late-onset Alzheimer's disease. These findings, which we hypothesize may reflect a more tau-dominant pathobiology in EOAD, challenge the universal diagnostic threshold paradigm and underscore the need for age-stratified interpretation of plasma biomarker results in both clinical and research settings. Prospective validation of age-stratified p-tau217 cutoffs, age-specific prognostic models, and corresponding interpretation frameworks is warranted.

## Supplementary Information

Supplementary Material 1: Supplementary Figure S1. Biomarker–clinical correlation heatmap in the total cognitive impairment cohort (circularity defense). Spearman rank correlation heatmap for all 505 CI participants (without AT(N)-based subsetting), mirroring the layout of Figure 2. This analysis addresses the potential circularity of using p-tau217 for both group definition and subsequent correlation by demonstrating that the direction, magnitude, and significance of key biomarker–clinical associations are preserved when the entire CI cohort is analyzed without diagnostic subgrouping.

Supplementary Material 2: Supplementary Figure S2. Biomarker–clinical correlation heatmaps for diagnostic subgroups. (A) Confirmed AD subgroup (*n =* 99). (B) Presumed AD subgroup (*n =* 227). Heatmaps display Spearman rank correlation coefficients between plasma biomarkers and clinical characteristics, identical in format to Figure 2. These subgroup-specific analyses verify that the correlation patterns observed in the consolidated AD continuum are consistent across the constituent subgroups.

Supplementary Material 3: Supplementary Figure S3. Complete age-stratified biomarker–clinical scatter plots. Comprehensive scatter plots with regression lines and 95% confidence bands for all biomarker–clinical variable pairs stratified by EO (<65 years, blue/purple) and LO ≥65 years, red/pink) within the AD continuum (S3A, Pages 1–7) and Non-AD CI (S3B, Pages 1–4). Each panel shows Spearman ρ, P value, and Fisher's Z-test P_diff for EO vs LO comparison. Marginal kernel density estimates are displayed for both groups. * *P* < 0.05.

Supplementary Material 4: Supplementary Figure S4. Multi-cutpoint sensitivity analysis and DeLong's test for age-stratified diagnostic performance. (A–B) Forest plots showing AUC with 95% CI for EO and LO groups at four age cutpoints (55, 60, 65, 70 years) in the ADNI cohort for p-tau217 and Aβ42/Aβ40. DeLong's P values shown as a separate column. (C–D) Summary tables of optimal cutoffs, AUC, sensitivity, and specificity for each age cutpoint and biomarker.

Supplementary Material 5: Supplementary Figure S5. Biomarker–clinical and biomarker–tau PET correlations in the ADNI validation cohort. (A) AD continuum proxy (Aβ+ subgroup): 4 clinical panels (MMSE, CDR-SB, NPI-Q Total, FAQ) and 4 tau PET SUVR panels (meta-temporal, entorhinal, hippocampus, amygdala). (B) Non-AD CI proxy (Aβ− MCI/DEM): same 8 panel layout. All panels show scatter plots with EO (blue circles) and LO (amber triangles), regression lines, 95% CI bands, marginal KDEs, and Fisher's Z-test for EO vs LO correlation difference.

Supplementary Material 6:Supplementary Table S1. Extended diagnostic performance metrics with predictive values. Sensitivity, specificity, positive predictive value (PPV), and negative predictive value (NPV) with bootstrap 95% confidence intervals (1,000 resamples) for the top-performing plasma biomarkers (p-tau217, p-tau181, p-tau217%, p-tau217/Aβ42, p-tau181/Aβ42, Aβ42/Aβ40, GFAP) against Aβ-PET status in the overall validation sub-cohort. Point estimates of Se and Sp correspond to the Youden-optimal cutoff reported in Table 2. PPV and NPV were computed using the observed Aβ+ prevalence (67.5%) in the PET sub-cohort. 

Supplementary Material 7: Supplementary Table S2. Complete Spearman rank correlation parameters between plasma biomarkers and clinical characteristics. Full correlation matrix for the AD continuum (*n=* 326) and Non-AD CI (*n =* 179) groups, including Spearman ρ, raw P values, and FDR-adjusted P values.

Supplementary Material 8: Supplementary Table S3. Age-stratified Spearman rank correlation parameters with Fisher's Z-test. EO and LO Spearman ρ, P values, and Fisher's Z P_diff for all biomarker–clinical pairs within the AD continuum and Non-AD CI groups.

Supplementary Material 9: Supplementary Table S4. Grey-zone sensitivity analysis, DeLong's test results, and diagnostic thresholds. Grey-zone analysis (<60 vs ≥70, excluding 60–70), DeLong's test comparing EO vs LO AUC in both cohorts, multi-cutpoint AUC data, and Youden-optimal thresholds for EO, LO, and overall groups.

Supplementary Material 10: Supplementary Table S5. ADNI longitudinal cognitive decline analysis. Baseline plasma p-tau217 as a predictor of annualized change in MMSE and CDR-SB, stratified by EO/LO and Aβ status.

Supplementary Material 11: Supplementary Table S6. Missing data analysis for the primary cohort. Panel A: missingness rates for key biomarker and clinical variables. Panel B: Little's MCAR test (χ²= 117.21, df = 24, *P* < 0.001), rejecting the MCAR assumption. Panel C: demographic and clinical comparison between participants with and without p-tau231 (the variable with the highest missingness, 55.5%), indicating a missing-at-random (MAR) mechanism attributable to clinical prioritization of biomarker testing in more severely affected patients.

Supplementary Material 12: Supplementary Table S7. Demographic and diagnostic performance characteristics of the ADNI external validation cohort. Baseline characteristics of 1,615 unique ADNI participants with available plasma biomarker data (Panel A), stratified by clinical diagnosis (CN, MCI, DEM) and age group (EO <65 years, LO ≥65 years). Age is presented as mean ± SD. Amyloid PET positivity defined as Centiloid ≥ 20.6. Panel B presents diagnostic performance of plasma p-tau217 (Lilly immunoassay) and Aβ42/Aβ40 (Fujirebio Lumipulse) against amyloid PET status for overall, EO, and LO groups. AUC = area under the ROC curve (95% CI, 1,000 bootstrap resamples); Se = sensitivity; Sp = specificity at the Youden-optimal cutoff

Supplementary Material 13: Supplementary Table S8. PET-confirmed AD–only sensitivity analysis of age-stratified biomarker–clinical correlations. Comparison of the eight headline early-onset (EO) versus late-onset (LO) biomarker–clinical correlation contrasts between the pre-specified full AD continuum (*n =* 326) and a sensitivity analysis restricted to PET-confirmed AD only (*n =* 99, all amyloid-PET positive; EO 37/LO 62). Spearman ρ; between-subgroup differences tested by Fisher's Z. Post-hoc power denotes the power of the PET-only sample sizes to detect the effect-size difference observed in the full continuum (α = 0.05, two-sided). Direction concordance is summarised across all computable pairs.

Supplementary Material 14: Supplementary Table S9. Formal age × biomarker interaction analysis and tau-related versus non-tau comparison. For each biomarker–clinical pair within the AD continuum (*n =* 326), an ordinary-least-squares model (standardized clinical ~ standardized biomarker × age group; EO onset <65 vs LO ≥65) was fitted and the standardized interaction coefficient extracted. Panel (a) compares tau-related markers (single-analyte tau plus tau/Aβ42 ratios) with non-tau markers (amyloid, GFAP, NfL) by interaction magnitude (Mann–Whitney U) and by the proportion of significant interactions (Fisher exact); panel (b) lists all interactions with 95% confidence intervals, raw P, and Benjamini–Hochberg FDR-adjusted P.

Supplementary Material 15: Supplementary Methods. Detailed plasma assay protocols, AT(N) classification criteria, and statistical methods.

## Data Availability

The primary cohort data are available from the corresponding author upon reasonable request. ADNI data are publicly available at adni.loni.usc.edu.

## Abbreviations

AD: Alzheimer’s disease
p-tau217: Phosphorylated tau-217
EOAD: Early-onset Alzheimer's disease
LOAD: Late-onset Alzheimer's disease
EO: Early-onset
LO: Late-onset
NC: Normal cognition
CI: Cognitive impairment
CDR: Clinical Dementia Rating
CDR-SB: Clinical Dementia Rating Sum of Boxes
MMSE: Mini-Mental State Examination
MoCA: Montreal Cognitive Assessment
ADL: Activities of Daily Living
NPI: Neuropsychiatric Inventory
CMAI: Cohen-Mansfield Agitation Inventory
ROC: Receiver operating characteristic
AUC: Area under the curve
APOE: Apolipoprotein E
Aβ: Amyloid-beta
PET: Positron emission tomography
SUVR: Standardized uptake value ratio
GFAP: Glial fibrillary acidic protein
NfL: Neurofilament light chain
FDR: False discovery rate
IQR: Interquartile range
STARD: Standards for Reporting Diagnostic Accuracy Studies
NIA-AA: National Institute on Aging–Alzheimer’s Association
ADNI: Alzheimer’s Disease Neuroimaging Initiative
DLB: Dementia with Lewy bodies
FAQ: Functional Activities Questionnaire
PPV: Positive predictive value
NPV: Negative predictive value
EDTA: Ethylenediaminetetraacetic acid
MCAR: Missing completely at random
