# Supplementary figures and images for: Age-dependent diagnostic and correlational architecture of multiplex plasma biomarkers in Alzheimer’s disease: a cross-ethnic, cross-platform validation study
Source: Alzheimers Res Ther. 2026 Jun 25;18:169. doi: 10.1186/s13195-026-02119-z (PMC13393608; doi:10.1186/s13195-026-02119-z)

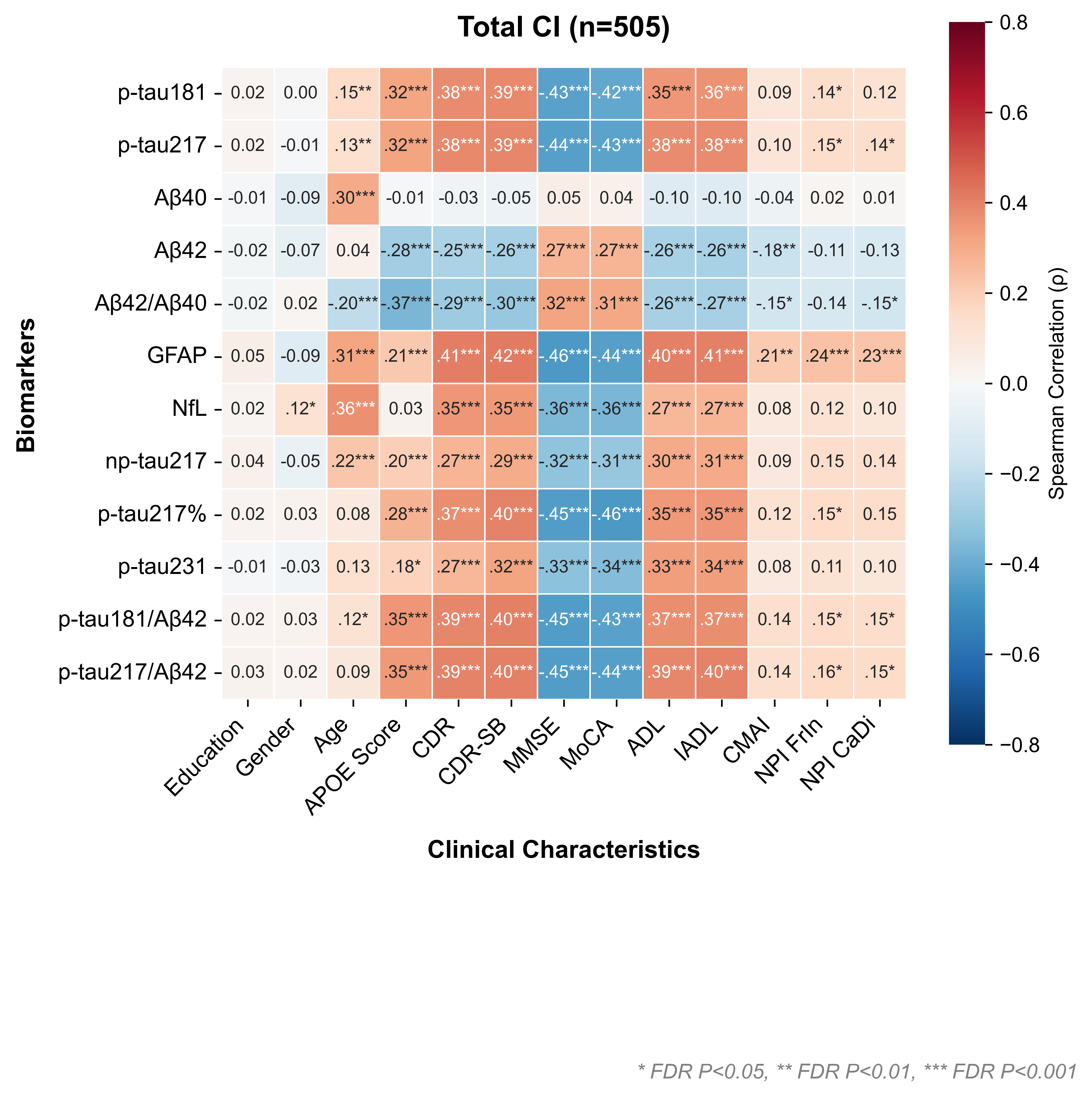

Supplement: Supplementary file 1 — Supplementary Material 1: Supplementary Figure S1. Biomarker–clinical correlation heatmap in the total cognitive impairment cohort (circularity defense). Spearman rank correlation heatmap for all 505 CI participants (without AT(N)-based subsetting), mirroring the layout of Figure 2. This analysis addresses the potential circularity of using p-tau217 for both group definition and subsequent correlation by demonstrating that the direction, magnitude, and significance of key biomarker–clinical associations are preserved when the entire CI cohort is analyzed without diagnostic subgrouping. [file 13195_2026_2119_MOESM1_ESM.tiff]

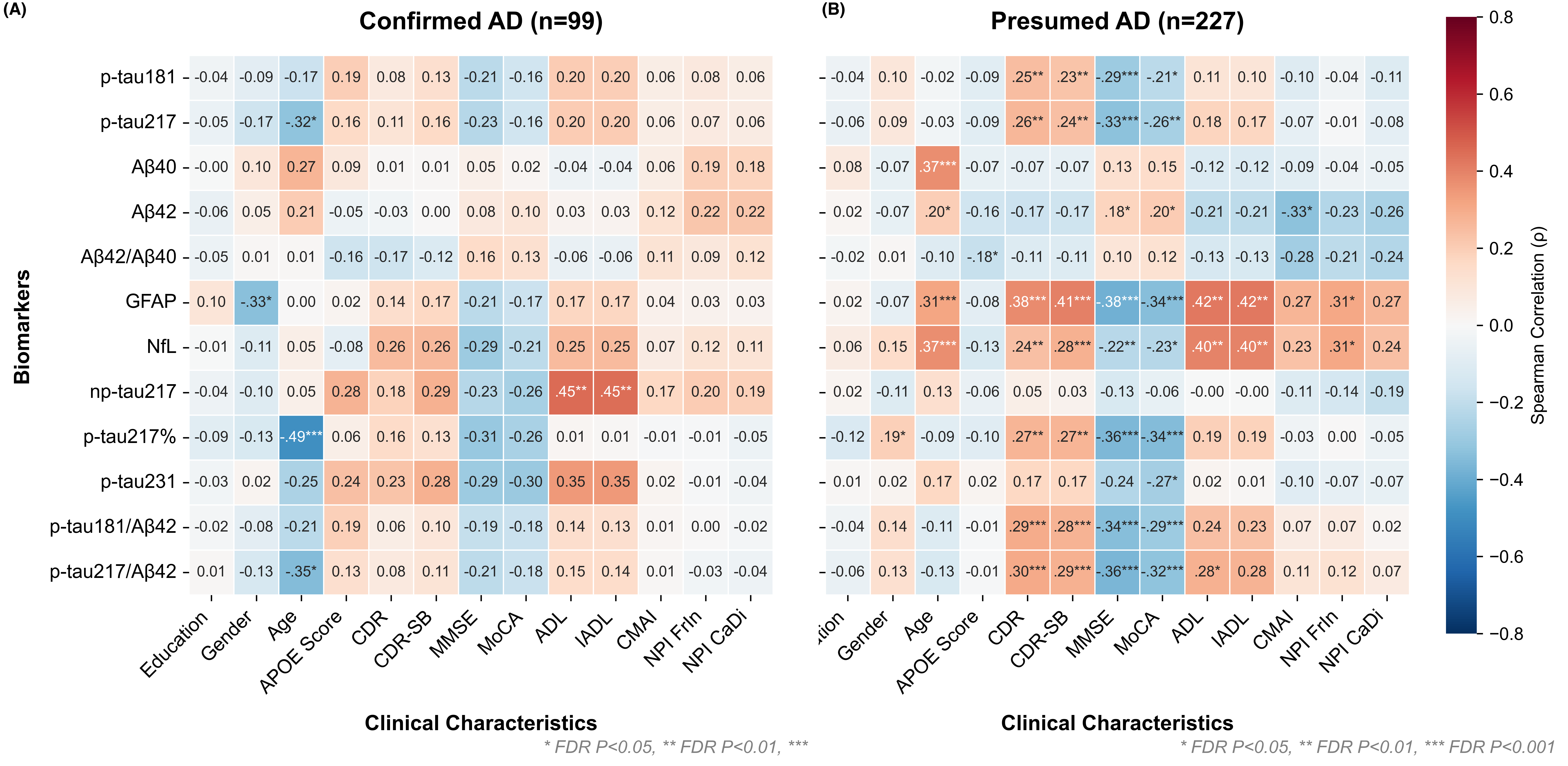

Supplement: Supplementary file 2 — Supplementary Material 2: Supplementary Figure S2. Biomarker–clinical correlation heatmaps for diagnostic subgroups. (A) Confirmed AD subgroup (n = 99). (B) Presumed AD subgroup (n = 227). Heatmaps display Spearman rank correlation coefficients between plasma biomarkers and clinical characteristics, identical in format to Figure 2. These subgroup-specific analyses verify that the correlation patterns observed in the consolidated AD continuum are consistent across the constituent subgroups. [file 13195_2026_2119_MOESM2_ESM.tiff]

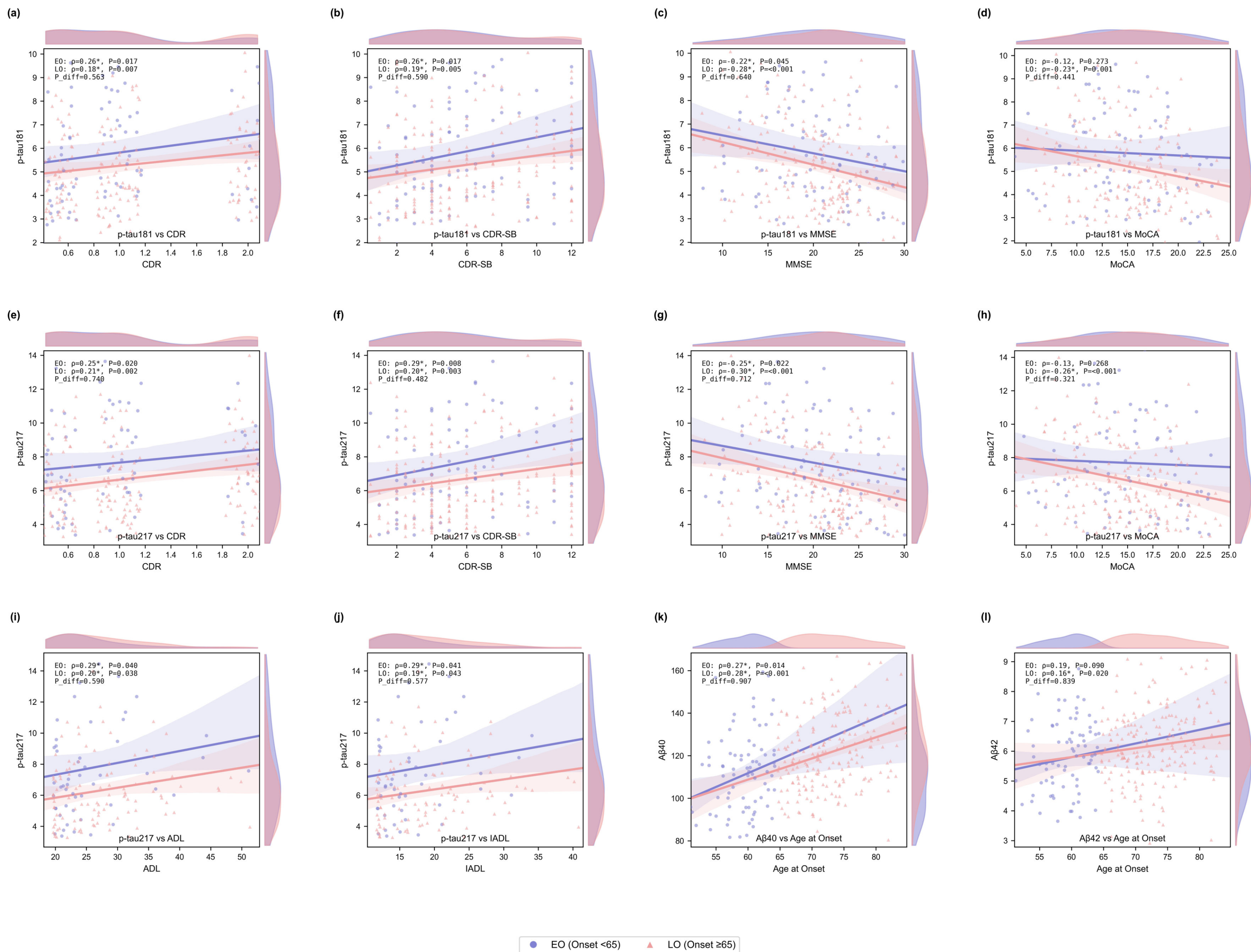

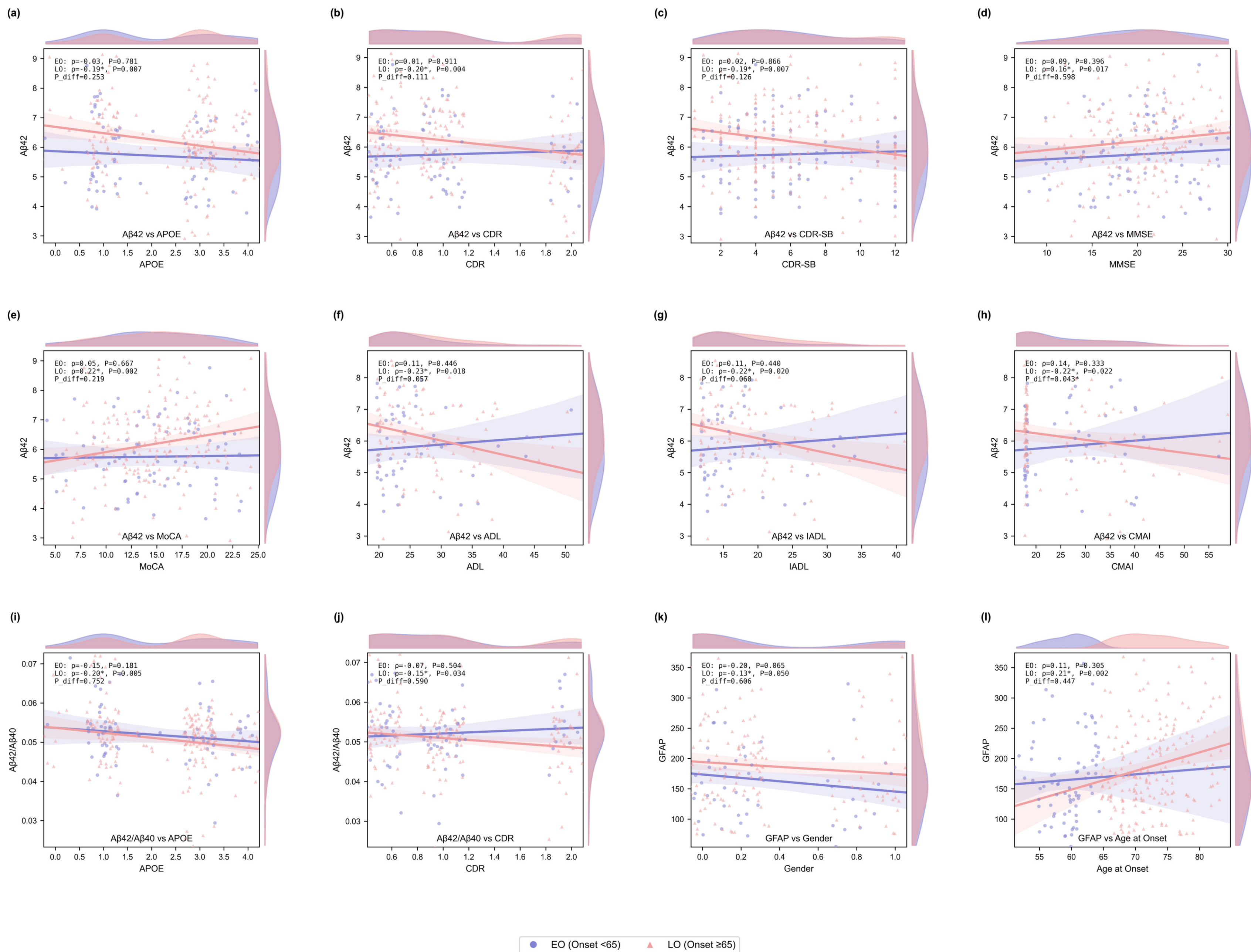

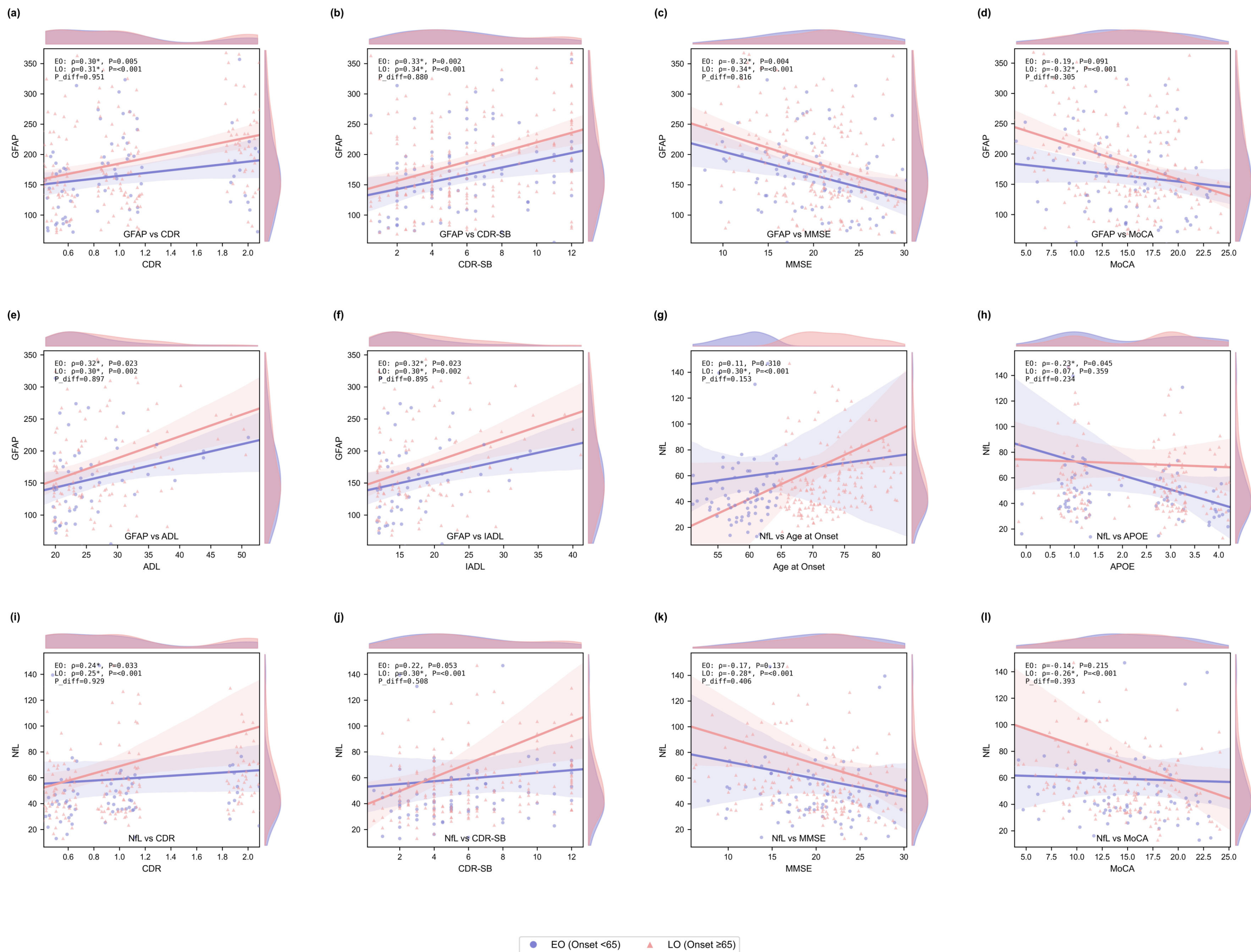

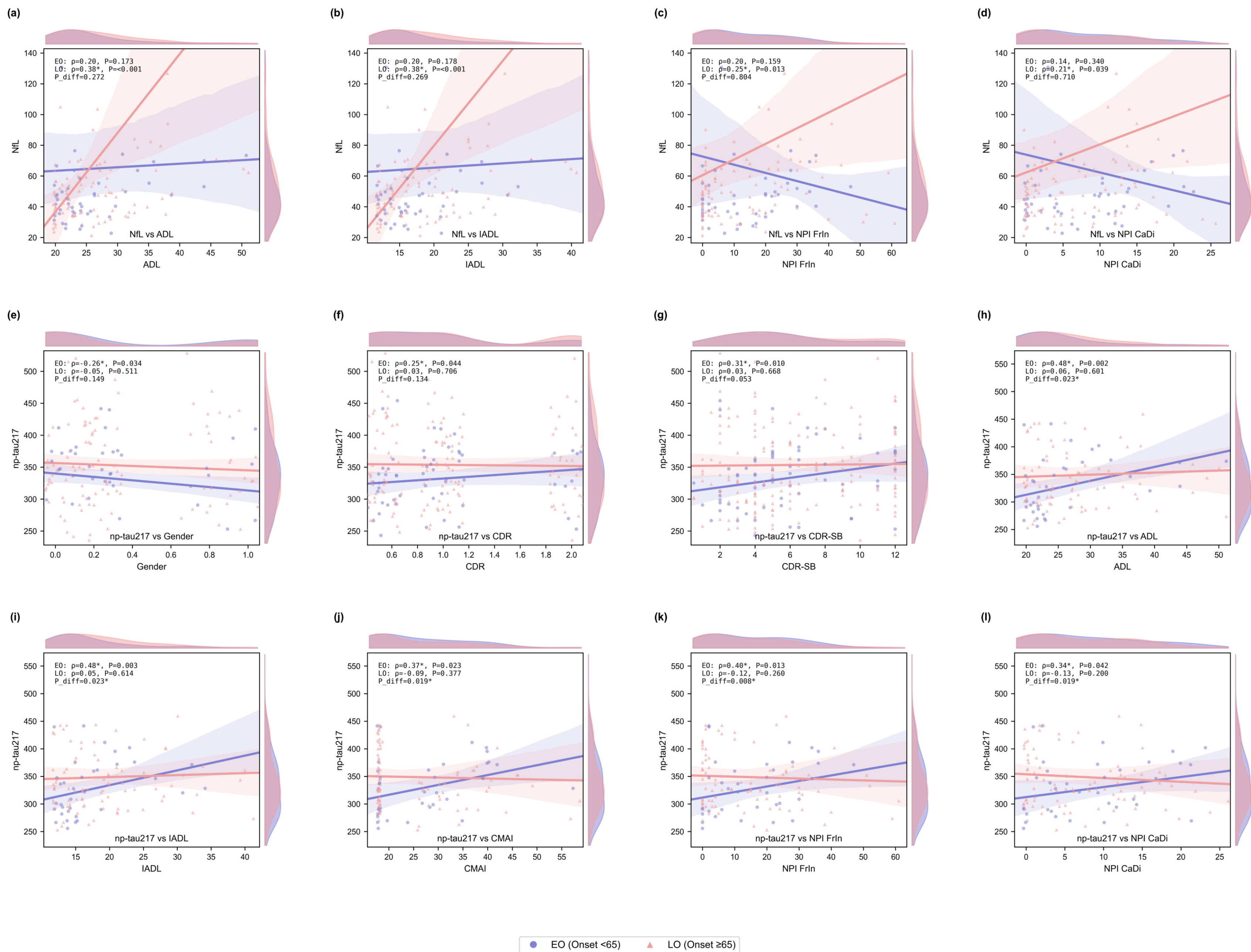

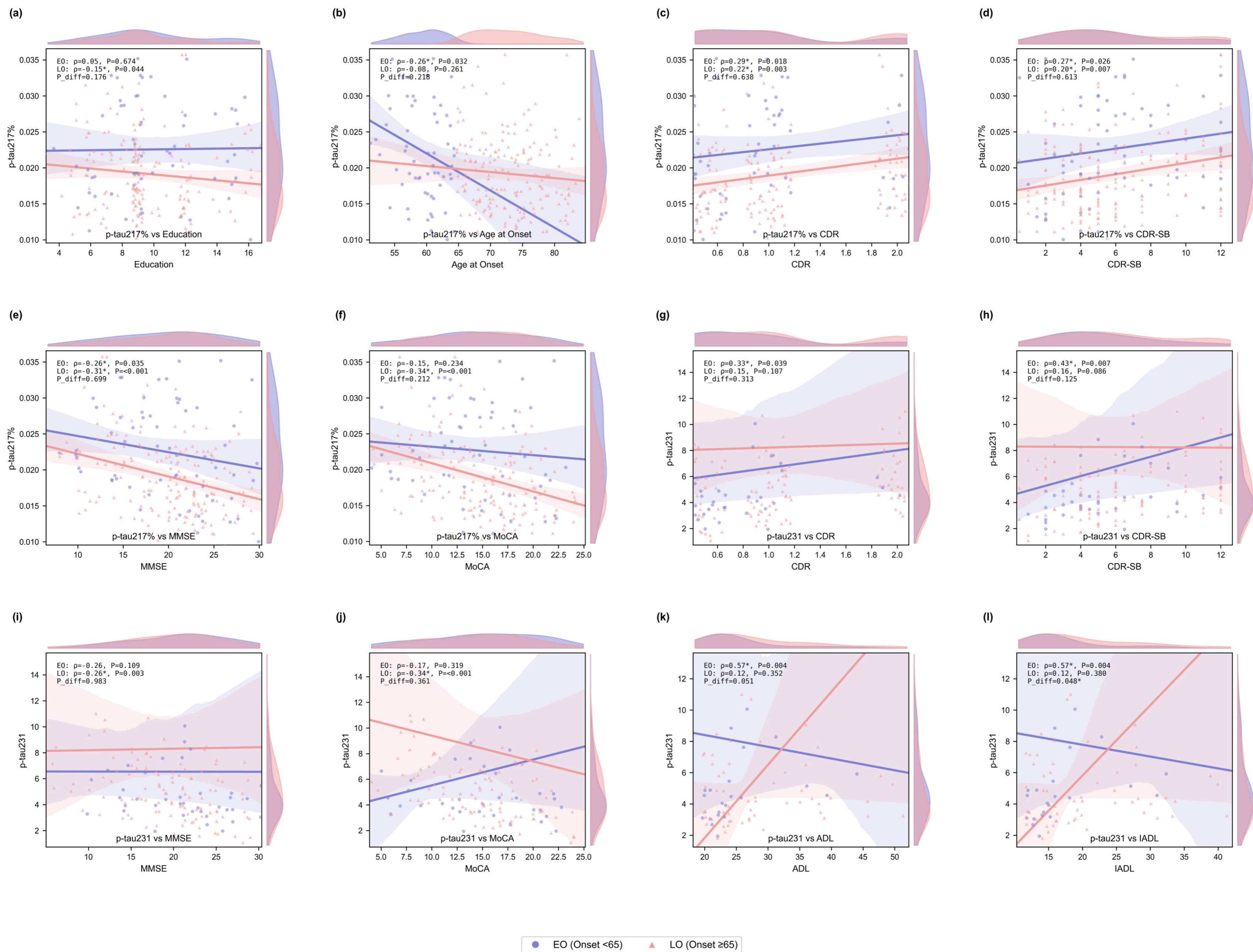

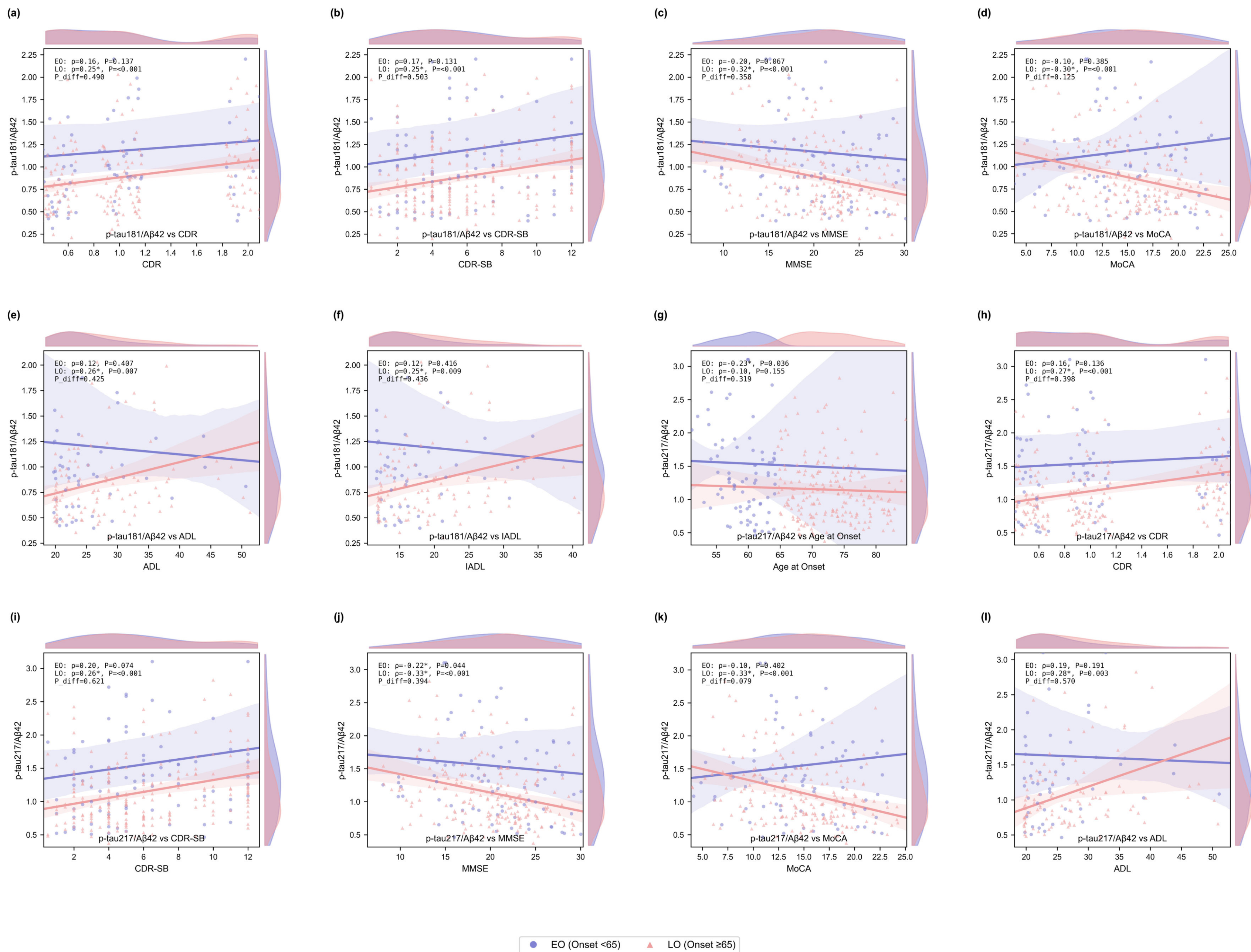

(a)

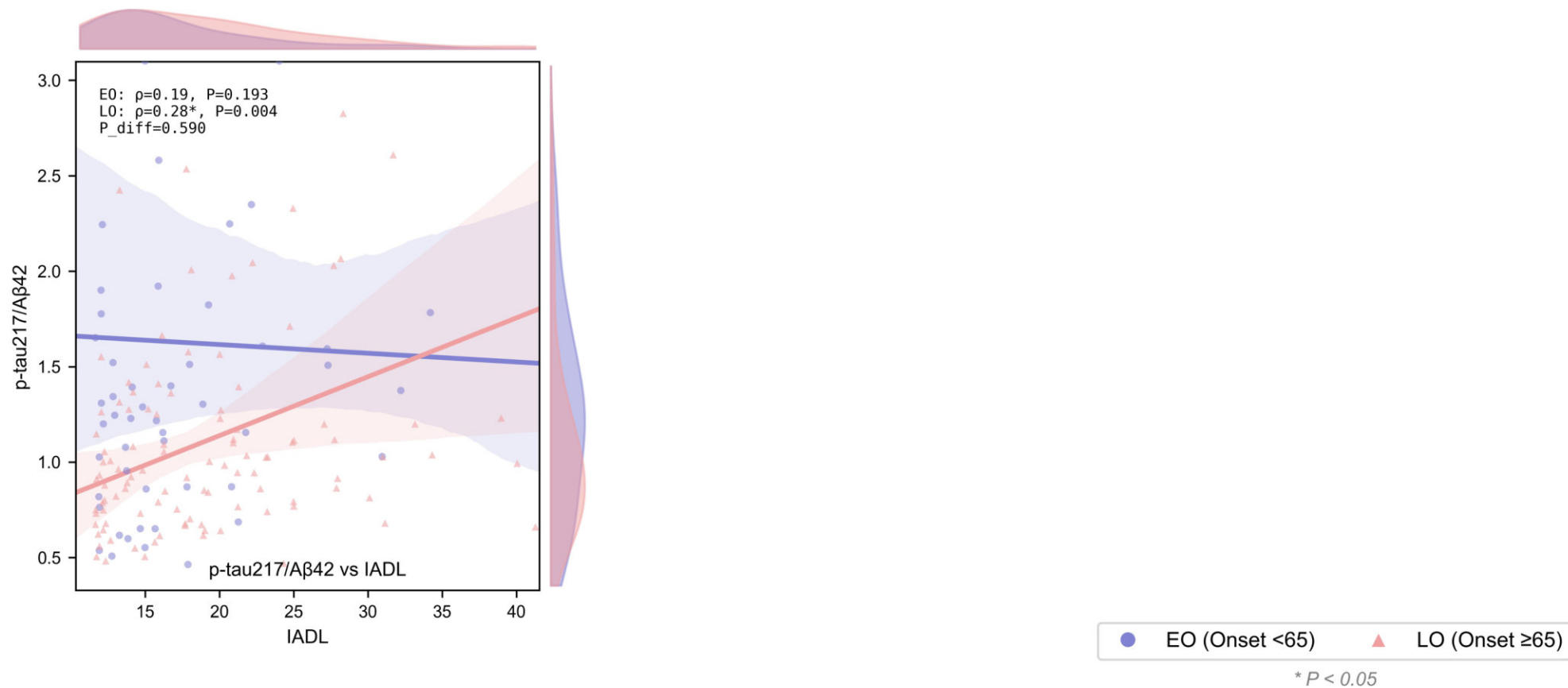

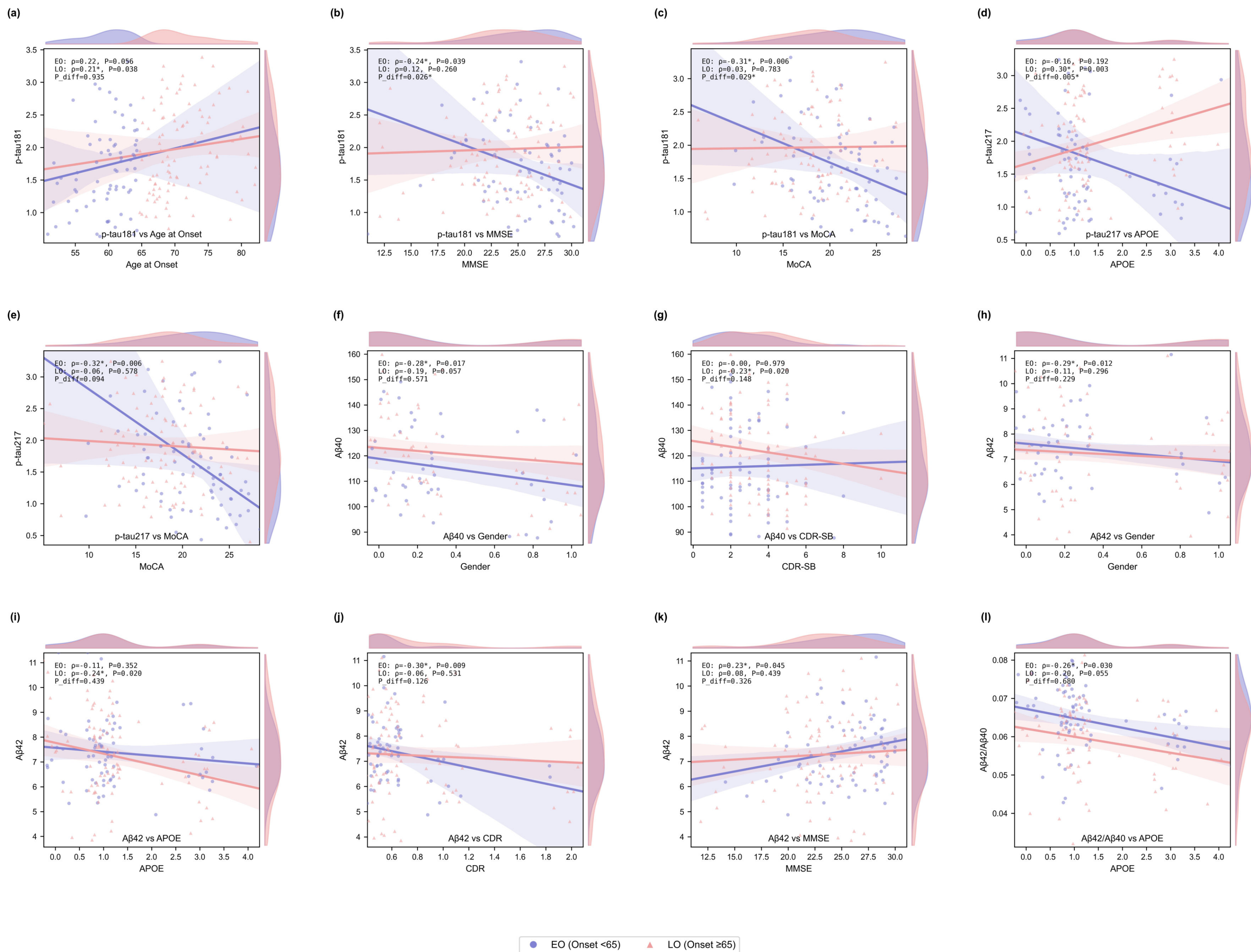

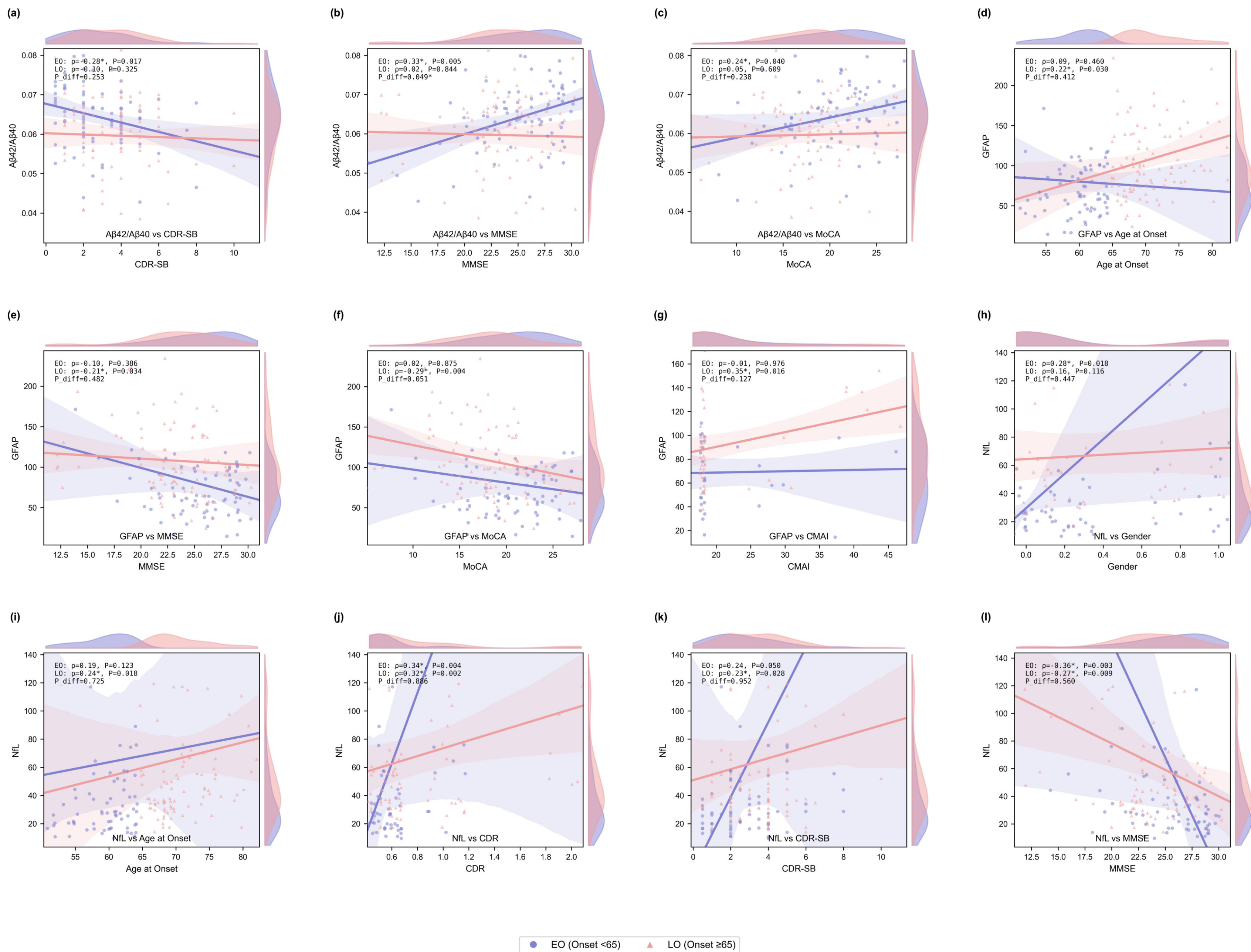

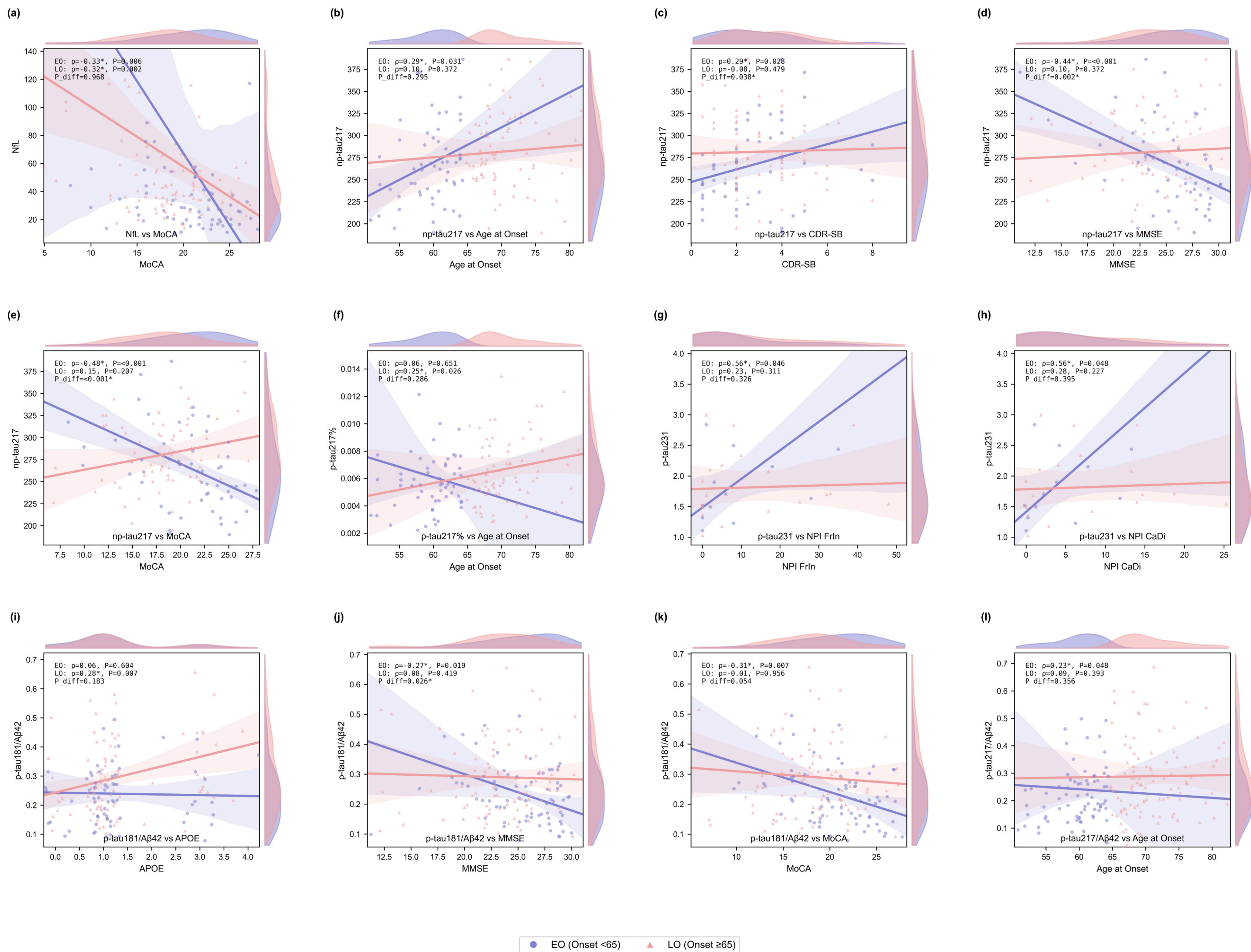

Supplementary Figure S3B\_Page4. Non-AD CI EO vs LO

(a)

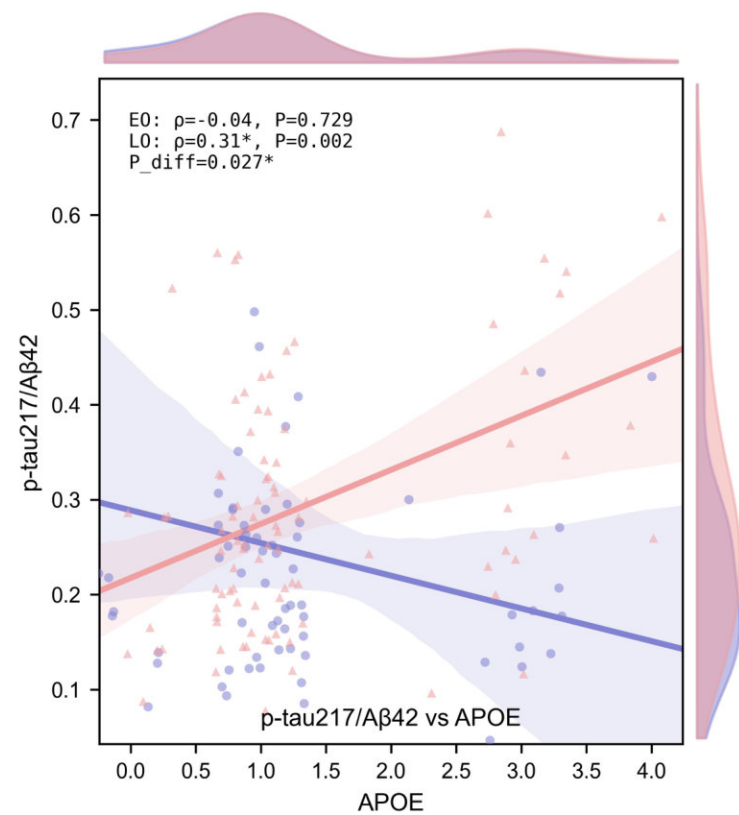

(b)

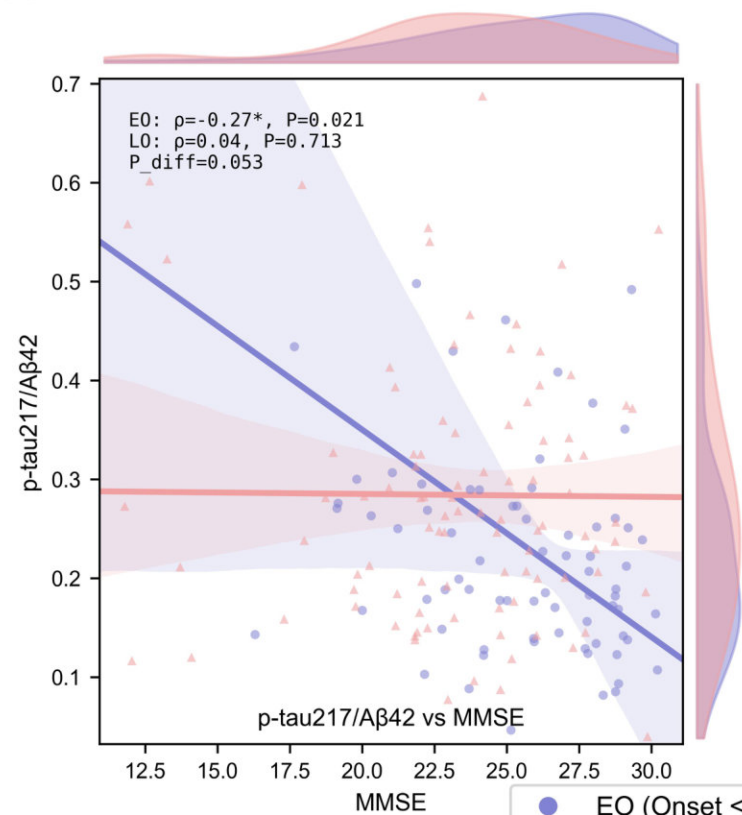

(c)

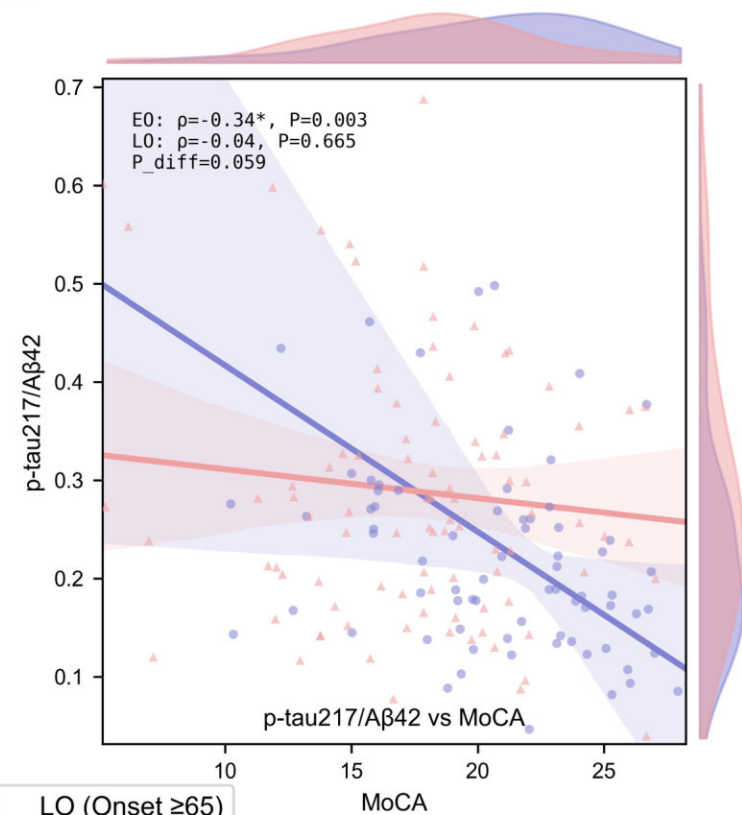

● EO (Onset <65)    ▲ LO (Onset ≥65)

\*  $P < 0.05$

Supplement: Supplementary file 3 — Supplementary Material 3: Supplementary Figure S3. Complete age-stratified biomarker–clinical scatter plots. Comprehensive scatter plots with regression lines and 95% confidence bands for all biomarker–clinical variable pairs stratified by EO (<65 years, blue/purple) and LO ≥65 years, red/pink) within the AD continuum (S3A, Pages 1–7) and Non-AD CI (S3B, Pages 1–4). Each panel shows Spearman ρ, P value, and Fisher's Z-test P_diff for EO vs LO comparison. Marginal kernel density estimates are displayed for both groups. * P < 0.05. [file 13195_2026_2119_MOESM3_ESM.pdf]

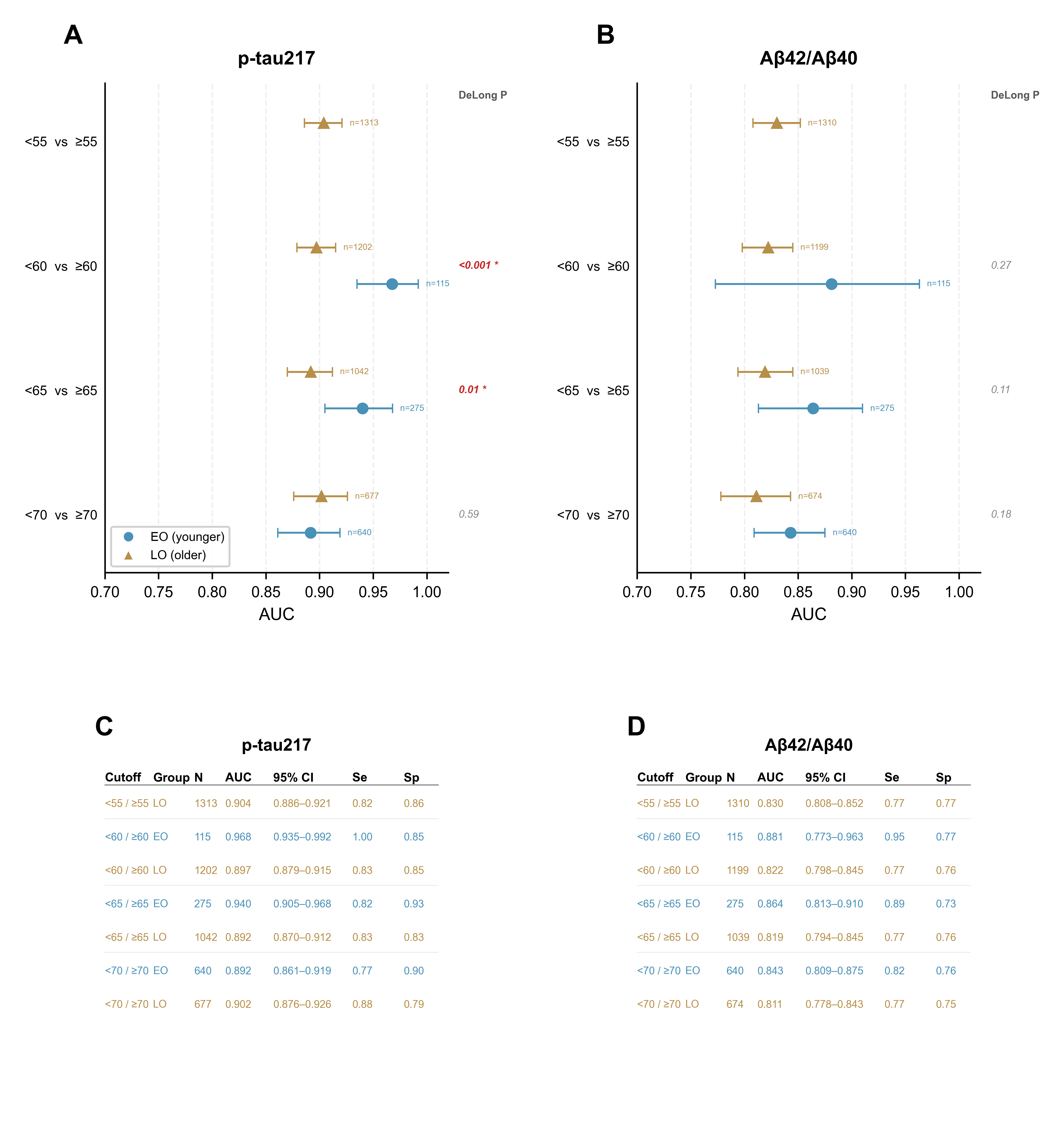

Supplement: Supplementary file 4 — Supplementary Material 4: Supplementary Figure S4. Multi-cutpoint sensitivity analysis and DeLong's test for age-stratified diagnostic performance. (A–B) Forest plots showing AUC with 95% CI for EO and LO groups at four age cutpoints (55, 60, 65, 70 years) in the ADNI cohort for p-tau217 and Aβ42/Aβ40. DeLong's P values shown as a separate column. (C–D) Summary tables of optimal cutoffs, AUC, sensitivity, and specificity for each age cutpoint and biomarker. [file 13195_2026_2119_MOESM4_ESM.tiff]

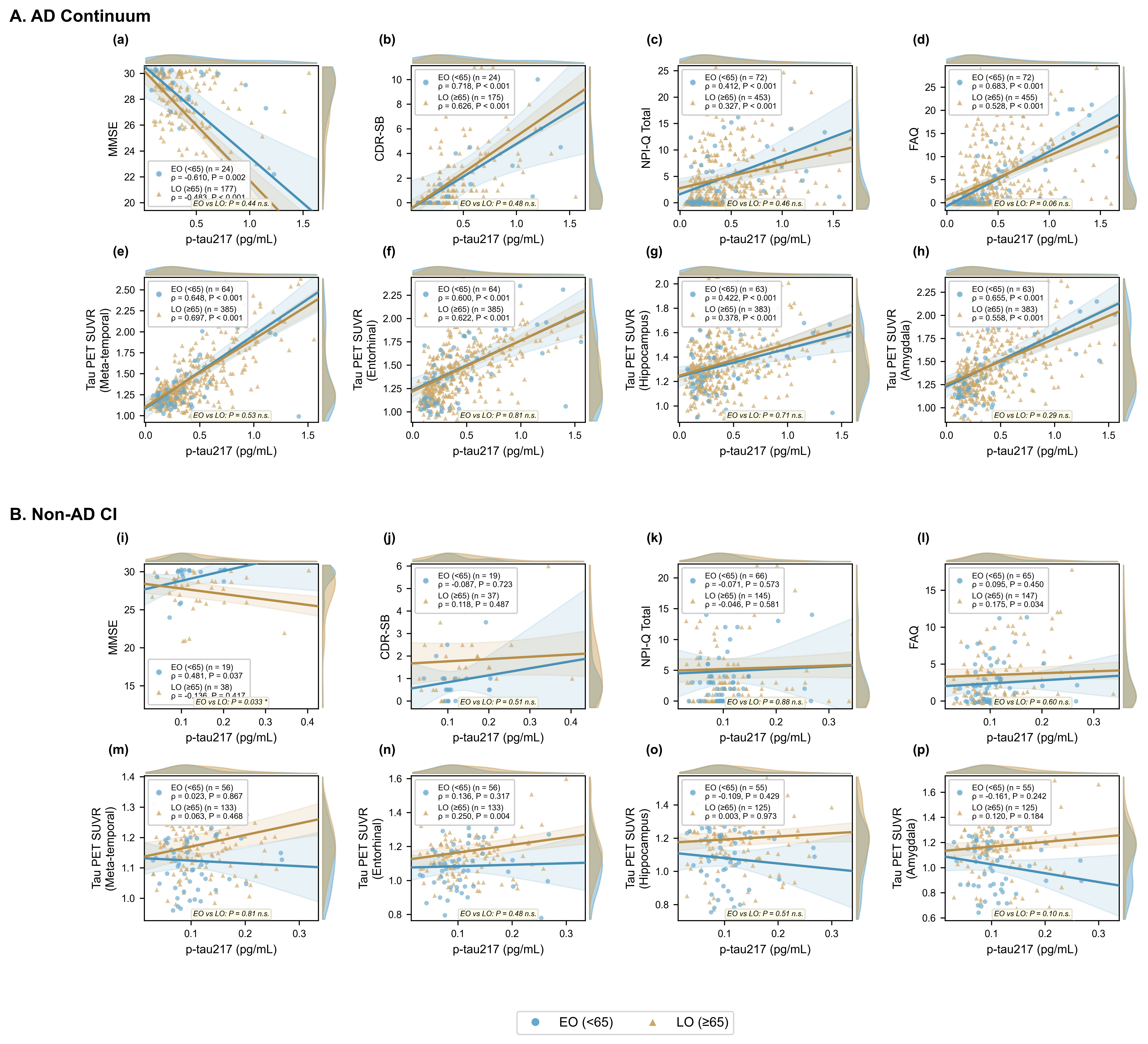

Supplement: Supplementary file 5 — Supplementary Material 5: Supplementary Figure S5. Biomarker–clinical and biomarker–tau PET correlations in the ADNI validation cohort. (A) AD continuum proxy (Aβ+ subgroup): 4 clinical panels (MMSE, CDR-SB, NPI-Q Total, FAQ) and 4 tau PET SUVR panels (meta-temporal, entorhinal, hippocampus, amygdala). (B) Non-AD CI proxy (Aβ− MCI/DEM): same 8 panel layout. All panels show scatter plots with EO (blue circles) and LO (amber triangles), regression lines, 95% CI bands, marginal KDEs, and Fisher's Z-test for EO vs LO correlation difference. [file 13195_2026_2119_MOESM5_ESM.tiff]
