# Supplementary material for: Age-dependent diagnostic and correlational architecture of multiplex plasma biomarkers in Alzheimer’s disease: a cross-ethnic, cross-platform validation study
Source: Alzheimers Res Ther. 2026 Jun 25;18:169. doi: 10.1186/s13195-026-02119-z (PMC13393608; doi:10.1186/s13195-026-02119-z)
Supplement: Supplementary file 15 — Supplementary Material 15: Supplementary Methods. Detailed plasma assay protocols, AT(N) classification criteria, and statistical methods. [file 13195_2026_2119_MOESM15_ESM.docx]

# Supplementary Methods

## S1. Detailed Exclusion Criteria

Participants were excluded if they had conditions that could interfere with behavioral and psychological symptom assessment or blood biomarker analyses. Specific exclusion criteria included: (1) psychiatric disorders such as schizophrenia or bipolar disorder; (2) other neurodegenerative diseases, including Parkinson's disease and dementia with Lewy bodies (DLB); (3) recent severe stroke; (4) severe cardiac, hepatic, or renal dysfunction, or malignant tumors; and (5) inability to complete key assessments (e.g., venous blood sampling, structural MRI, or the full neuropsychological and behavioral battery). Participants with severe dementia (CDR > 2) were not enrolled, as they were unable to cooperate with the comprehensive evaluations required by the study protocol.

## S2. Clinical Evaluation: Additional Details

Comprehensive demographic data were collected for all participants, including age at symptom onset (or age at enrollment for NC participants), sex, years of education, APOE genotype, occupation, and medical history. All participants underwent standardized neuropsychological testing administered by uniformly trained professionals in a controlled environment. Clinical assessors performing neuropsychological and behavioral evaluations were blinded to the participants' biomarker results and PET imaging findings.

The CMAI-cs is an 18-item caregiver-rated scale assessing agitated behaviors across four domains: physical aggressive, physical non-aggressive, verbal aggressive, and other behaviors. The scale showed adequate psychometric properties in our cohort (Cronbach's α = 0.723, ICC = 0.975; detailed validation reported separately [manuscript in preparation]).

The ADL scale comprises two subscales: Basic Activities of Daily Living (BADL) and Instrumental Activities of Daily Living (IADL).

## S3. Blood Sample Collection and Processing Protocol

To limit diurnal variation, fasting venous blood was obtained in the morning. Before sampling, participants were asked to avoid strenuous exercise, and anyone with an active infection or possible drug interference was screened beforehand. Samples were drawn into EDTA-coated tubes. Tubes and reagents came from a single manufacturing batch throughout the study to limit batch effects. At least two tubes were obtained per participant for duplicate measurement and backup. Each tube was gently inverted right after collection to mix the sample evenly and prevent hemolysis. Samples stood at room temperature for 30–60 minutes to allow plasma separation, and were then centrifuged at 2,000 × g for 15 minutes at 4°C. The plasma supernatant was aliquoted and stored immediately at −80°C. Before analysis, aliquots were thawed on ice and went through only a single freeze-thaw cycle. All biomarker assays were performed by Nanjing Vazyme Medical Technology Co., Ltd.

## S4. Biomarker Assay Kit Catalog Numbers and Assay Principle

### Catalog Numbers

Plasma biomarkers were measured using the following matched assay kits on the Shine i2910 Chemiluminescence Immunoassay Analyzer (Vazyme Medical Technology, China):

| Biomarker | Catalog No. |
| --- | --- |
| Aβ42 | M2705CB |
| Aβ40 | M1705CB |
| p-tau181 | M3705CB |
| p-tau217 | M4705CB |
| p-tau231 | N2701CB |
| np-tau217 | P2701CB |
| NfL | M6705CB |
| GFAP | M5705CB |

The derived metric p-tau217% was calculated as the ratio of phosphorylated tau 217 to total tau 217: p-tau217 / (p-tau217 + np-tau217).

### Assay Principle

These kits use direct chemiluminescence detection in a sandwich immunoassay format. Magnetic particles are coated with monoclonal antibodies against each target biomarker, while a second monoclonal antibody labeled with acridinium ester acts as the detection conjugate. After incubation, antibody–antigen–antibody complexes form during the immunoreaction. Adding pre-trigger and trigger solutions causes these complexes to emit light, which the analyzer captures. The resulting relative light units (RLUs) are directly proportional to the analyte concentration in each sample.

### Quality Control

All laboratory personnel performing the assays were blinded to the clinical diagnoses and group allocations. Assay performance was monitored with internal quality controls; intra-assay and inter-assay coefficients of variation (CVs) were kept at < 10% for all measured biomarkers. Sample measurements falling below the lower limit of quantification (LLoQ) were excluded from the analysis, which accounts for a small proportion of missing values in the final dataset. p-tau231 had a substantially higher rate of missing data than other analytes because it was not part of the routine biomarker panel and was assayed only from residual plasma aliquots after all primary measurements had been completed; in many cases, the remaining sample volume was insufficient for this additional assay.

## S5. Aβ-PET Image Acquisition and Preprocessing

Amyloid PET was acquired with the ¹⁸F-florbetapir (AV-45) radiotracer. Raw PET data were preprocessed with correction for attenuation, scatter, random events, and motion. Partial volume correction (PVC) was applied where needed to reduce signal spillover. Processed images were read primarily by visual interpretation, supported by standardized uptake value ratio (SUVr) data.

Two trained nuclear medicine physicians and dementia experts independently reviewed the scans. In cases of discrepancy, a third senior nuclear medicine physician was consulted to reach a consensus. Inter-rater agreement between the two primary readers was excellent (Cohen's κ = 0.92, 95% CI: 0.86–0.98; concordance rate = 96.6%); all discrepancies were resolved by the third reader, yielding a final consensus classification for each scan.

An ¹⁸F-florbetapir PET scan was classified as "amyloid PET-positive" if at least two distinct cortical regions exhibited tracer uptake, or if a single cortical region demonstrated significantly pronounced uptake.

### Assessment of Neurodegeneration

Neurodegeneration (N) status was assessed using structural MRI. The presence of brain atrophy was independently evaluated by two experienced specialists (one board-certified neurologist and one board-certified neuroradiologist) through visual inspection. All cognitively impaired participants (CDR ≥ 0.5) enrolled in this study were confirmed to have MRI evidence of brain atrophy (N+).

### Definitions of Cognitive Status

- **Normal cognition (NC):** Global CDR score of 0 with no subjective cognitive complaints.
- **Cognitive impairment (CI):** Global CDR score ≥ 0.5, encompassing both MCI and dementia stages.

## S6. ADNI External Validation: Additional Methodological Details

Data were obtained from the Alzheimer's Disease Neuroimaging Initiative (ADNI; adni.loni.usc.edu). A total of 1,615 ADNI participants with available plasma biomarker data were included (847 CN, 579 MCI, 186 DEM). Plasma p-tau217 was measured using the Lilly immunoassay, and plasma Aβ42/Aβ40 was measured using the Fujirebio Lumipulse platform. Amyloid PET positivity was defined as Centiloid ≥ 20.6. Tau PET SUVRs were obtained from the ADNI PET Core using ¹⁸F-flortaucipir.

Because ADNI does not record age at symptom onset, participants were stratified into EO (baseline age < 65 years, n = 335) and LO (baseline age ≥ 65 years, n = 1,280) groups using baseline age as a proxy. ROC analyses were performed on all participants with concurrent plasma biomarker and amyloid PET data (n = 1,317 for p-tau217; n = 1,314 for Aβ42/Aβ40). DeLong's test was used to compare EO vs LO AUCs within each cohort. Sensitivity analyses were performed using multiple age cutpoints (55, 60, 65, and 70 years). A grey-zone sensitivity analysis was conducted by excluding participants aged 60–70 years. Post-hoc statistical power for the primary cohort EO vs LO AUC comparison was estimated using the normal approximation for independent AUCs.

A longitudinal analysis was also performed using ADNI subjects with both baseline plasma p-tau217 and follow-up cognitive assessments (MMSE, CDR-SB). Annualized rates of change (Δscore/year) were computed, and Spearman correlations between baseline p-tau217 and cognitive decline rates were compared between EO and LO subgroups. To assess cross-ethnic generalizability, diagnostic performance was evaluated in the ADNI Asian subgroup separately.
